# Mycofactocin Is Associated with Ethanol Metabolism in Mycobacteria

**DOI:** 10.1128/mBio.00190-19

**Published:** 2019-05-21

**Authors:** Gopinath Krishnamoorthy, Peggy Kaiser, Laura Lozza, Karin Hahnke, Hans-Joachim Mollenkopf, Stefan H. E. Kaufmann

**Affiliations:** a Department of Immunology, Max Planck Institute for Infection Biology, Berlin, Germany; b Core Facility Microarray/Genomics, Max Planck Institute for Infection Biology, Berlin, Germany; c Hagler Institute for Advanced Study at Texas A&M University, College Station, Texas, USA; Washington University in St. Louis School of Medicine

**Keywords:** *Mycobacterium tuberculosis*, ethanol oxidation, mycofactocin, pyrroloquinoline quinone, redox cofactor, ribosomally synthesized and posttranslationally modified peptides

## Abstract

Mycofactocin (MFT) belongs to the class of ribosomally synthesized and posttranslationally modified peptides conserved in many *Actinobacteria*. Mycobacterium tuberculosis assimilates cholesterol during chronic infection, and its *in vitro* growth in the presence of cholesterol requires most of the MFT biosynthesis genes (*mftA*, *mftB*, *mftC*, *mftD*, *mftE*, and *mftF*), although the reasons for this requirement remain unclear. To identify the function of MFT, we characterized MFT biosynthesis mutants constructed in Mycobacterium smegmatis, M. marinum, and M. tuberculosis. We found that the growth deficit of *mft* deletion mutants in medium containing cholesterol—a phenotypic basis for gene essentiality prediction—depends on ethanol, a solvent used to solubilize cholesterol. Furthermore, functionality of MFT was strictly required for growth of free-living mycobacteria in ethanol and other primary alcohols. Among other genes encoding predicted MFT-associated dehydrogenases, *MSMEG_6242* was indispensable for M. smegmatis ethanol assimilation, suggesting that it is a candidate catalytic interactor with MFT. Despite being a poor growth substrate, ethanol treatment resulted in a reductive cellular state with NADH accumulation in M. tuberculosis. During ethanol treatment, *mftC* mutant expressed the transcriptional signatures that are characteristic of respirational dysfunction and a redox-imbalanced cellular state. Counterintuitively, there were no differences in cellular bioenergetics and redox parameters in *mftC* mutant cells treated with ethanol. Therefore, further understanding of the function of MFT in ethanol metabolism is required to identify the cause of growth retardation of MFT mutants in cholesterol. Nevertheless, our results establish the physiological role of MFT and also provide new insights into the specific functions of MFT homologs in other actinobacterial systems.

## Abstract (precis)

Tuberculosis is caused by Mycobacterium tuberculosis, and the increasing emergence of multidrug-resistant strains renders current treatment options ineffective. Although new antimycobacterial drugs are urgently required, their successful development often relies on complete understanding of the metabolic pathways—e.g., cholesterol assimilation—that are critical for persistence and for pathogenesis of M. tuberculosis. In this regard, mycofactocin (MFT) function appears to be important because its biosynthesis genes are predicted to be essential for M. tuberculosis *in vitro* growth in cholesterol. In determining the metabolic basis of this genetic requirement, our results unexpectedly revealed the essential function of MFT in ethanol metabolism. The metabolic dysfunction thereof was found to affect the mycobacterial growth in cholesterol which is solubilized by ethanol. This knowledge is fundamental in recognizing the bona fide function of MFT, which likely resembles the pyrroloquinoline quinone-dependent ethanol oxidation in acetic acid bacteria exploited for industrial production of vinegar.

## INTRODUCTION

**FIG 1:**
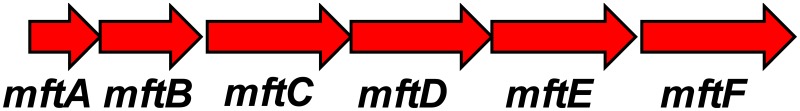
Genomic organization of *mft* genes in M. tuberculosis H37Rv and M. smegmatis mc^2^155. The schematic illustration (not drawn to scale) presents the gene names corresponding to the following *mft* ortholog locus numbers and functional annotations: *mftA*, *Rv0691A*/*MSMEG_1421*, precursor peptide; *mftB*, *Rv0692*/*MSMEG_1422*, MftA chaperone; *mftC*, *Rv0693*/*MSMEG_1423*, radical S-adenosylmethionine maturase; *mftD*, *Rv0694*/*MSMEG_1424*, flavin/heme dehydrogenase; *mftE*, *Rv0695*/*MSMEG_1425*, creatinine aminohydrolase family protein; *mftF*, *Rv0696*/*MSMEG_1426*, glycosyltransferase. *mftB*, *mftC*, *mftD*, *mftE*, and *mftF* are identified as essential for M. tuberculosis *in vitro* growth in cholesterol (20, 21).

Ribosomally synthesized and posttranslationally modified peptides (RiPPs) are a group of structurally distinct and functionally diverse natural products (1, 2), which include mycofactocin (MFT), resembling pyrroloquinoline quinone (PQQ) cofactor and bacteriocin (3). MFT biosynthesis appears to be conserved in many *Actinobacteria*, including Mycobacterium tuberculosis, the causative agent of human tuberculosis, which remains a global health threat (4). MFT biosynthesis is predicted to comprise a six-gene ensemble (*mft* genes): *mftA* and *mftB* encode the precursor peptide and its chaperone, respectively, and *mftC*, *mftD*, *mftE*, and *mftF* encode products that modify MftA or are functionally associated with the MftA-derived end product(s) or both (3) (Fig. 1). In recent years, the catalytic roles of MftC and MftE in the initial steps of MFT synthesis have been elucidated (5–9). Moreover, the possibility of identifying novel MFT-derived metabolite/s has been suggested, with the synthesis of these metabolites probably involving extensive posttranslational modification, in a stepwise or concerted action, similar to other RiPPs (1–3, 5–9).

RiPPs share many structural and enzymatic similarities with nonribosomal peptides (NRPs), except for the polyketide-derived moieties that are ubiquitous in NRPs (10). Exochelin and mycobactin are notable mycobacterial NRPs which are vital for iron acquisition and pathogenesis of M. tuberculosis (11, 12). Unlike NRPs, the significance of RiPPs such as MFT in mycobacteria is still elusive, although MFT is predicted to function as a cofactor, similar to PQQ, for its nicotinoprotein redox partners (3, 13). PQQ is a redox cofactor that supports the functions of several quinoproteins, including ethanol (EtOH) and aldehyde dehydrogenases catalyzing the oxidation of ethanol to acetic acid with acetaldehyde as a reaction intermediate (14–18). Besides the possibility of acting as an analog of PQQ and catalyzing ethanol oxidation in mycobacteria, MFT could have other cellular functions: (i) products encoded by *mft* genes have been suggested to be part of the electron transport chain, which is vital for M. tuberculosis survival under both replicating and nonreplicating conditions (19); (ii) independent saturated transposon mutagenesis studies (20, 21) have predicted the essentiality of most of the *mft* genes for *in vitro* growth in cholesterol—solubilized in ethanol—a carbon source required for host adaptation of M. tuberculosis during infection (22–24). While data from the studies mentioned above strongly imply the role of MFT in M. tuberculosis pathogenesis, the precise nature of the biochemical function of MFT in M. tuberculosis metabolism, particularly in cholesterol catabolism, remains unclear. Accordingly, exploring the conditions under which *mft* genes are essential for *in vitro* growth of M. tuberculosis in cholesterol could uncover the bona fide function(s) of MFT. Consequently, insights into the MFT-associated pathway(s) for potential therapeutic intervention could be provided and could light shed on the functions of MFT homologs conserved in many other bacterial species.

In this study, using genetic and transcriptomic methods, we revealed that the essentiality of *mft* genes for growth in cholesterol is dependent on the presence of ethanol in M. smegmatis, M. marinum, and M. tuberculosis. We showed further that disruption of *mft* genes affects the mycobacterial growth in ethanol. These findings have implications for respiration and redox regulation during mycobacterial growth/adaptation in ethanol.

## RESULTS

### MFT gene essentiality for M. smegmatis growth in cholesterol is ethanol dependent.

**FIG 2:**
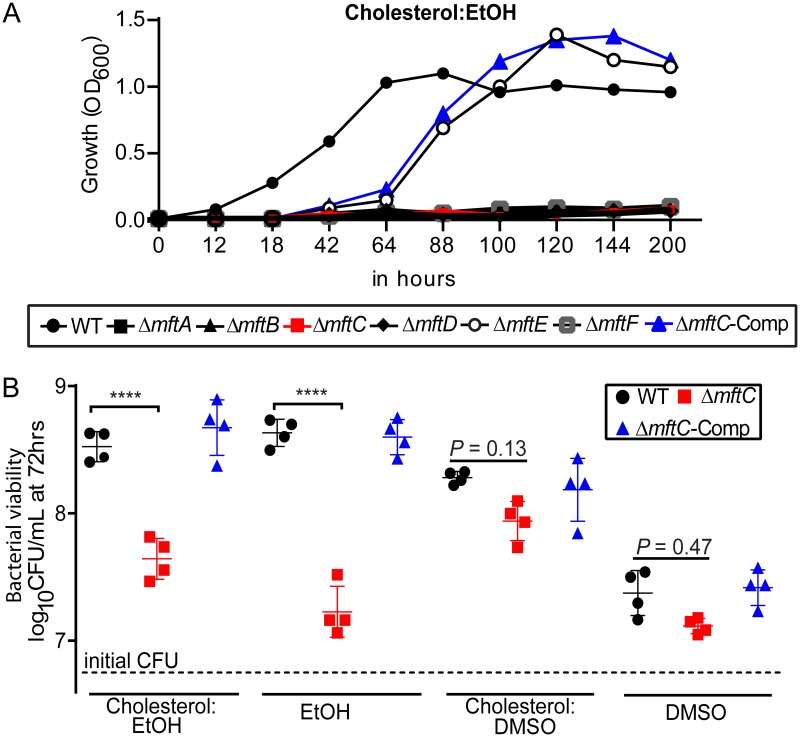
Ethanol limits the growth of *mft* mutants in medium containing cholesterol. (A) Growth of *mft* mutants and wild-type (WT) strains in liquid medium containing ethanol (1% [vol/vol])-solubilized 0.01% cholesterol (Cholesterol:EtOH) as the sole carbon source. Growth was monitored by measuring absorbance at 600 nm (reported as OD_600_). Data are representative of results from two independent experiments. (B) CFU were enumerated from growth in medium containing cholesterol:EtOH or cholesterol:DMSO (solubilized in hot DMSO). Respective solvent controls were included. Total numbers of CFU per milliliter (means ± standard deviations) were determined from four independent experiments performed in duplicate. Data presented in this panel were chosen to indicate altered growth rates of bacterial strains during the late-exponential-growth phase (at 72 h). Bacterial counts determined during the course of growth (up to 120 h) are presented in Fig. S2B and C. ****, *P < *0.001 (one-way analysis of variance [ANOVA]/Tukey’s multiple-comparison test on log-transformed data).

To determine the essentiality of *mft* genes for the growth of M. smegmatis in cholesterol, single in-frame deletion mutants of all *mft* genes (*mft* mutants) were constructed and characterized (see Fig. S1 in the supplemental material; see also Table S1 in the supplemental material). As expected, none of the *mft* mutants—except strain Δ*mftE*, which exhibited delayed growth—grew in minimal medium containing cholesterol (0.01% [wt/vol]) as the sole carbon source with hot ethanol (1% [vol/vol]) as the solvent (cholesterol and EtOH [cholesterol:EtOH]) (Fig. 2A). In contrast, the growth rates of all strains in glycerol-supplemented medium were comparable (Fig. S2A). Given the key role of MftC in the initial steps of MFT biosynthesis (3, 5–9) and the near-identical phenotypes of *mft* mutants, the Δ*mftC* strain was chosen for further analyses. Genetic complementation of strain Δ*mftC* with a full-length copy of *mftC* at the *attB* site, referred to as strain Δ*mftC*-Comp, reversed the growth defect, albeit with a delay, in cholesterol:EtOH. Thus, disruption of any of the *mft* genes resulted in impaired growth of M. smegmatis in cholesterol:EtOH. Subsequently, to interrogate whether the growth limitation of strain Δ*mftC* was ethanol dependent, the growth of strain Δ*mftC* was assessed in medium containing cholesterol solubilized in hot dimethyl sulfoxide (cholesterol-dimethyl sulfoxide [cholesterol:DMSO]). Strikingly, unlike the results seen with cholesterol:EtOH, there was no significant growth retardation in cholesterol:DMSO, although a minor deficit in strain Δ*mftC* growth in DMSO-containing medium in comparison with the growth of wild-type strain was noted (Fig. 2B; see also Fig. S2B and C). Thus, ethanol limits the growth of strain Δ*mftC* in cholesterol:EtOH.

10.1128/mBio.00190-19.1 FIG S1 Construction and genotypic characterization of mutant derivatives of M. smegmatis and M. tuberculosis. The target and flanking genes are shown as solid arrows (not drawn to scale). For Southern blot analyses, chromosomal DNA from the wild-type (WT) strains and PCR-confirmed mutants were digested with specific restriction enzymes. Expected fragment sizes (indicated in base pairs) from WT and mutant strains were detected using PCR-generated probes (solid arrows in red). Download FIG S1, TIF file, 1.8 MB. Copyright © 2019 Krishnamoorthy et al. 2019 Krishnamoorthy et al. This content is distributed under the terms of the Creative Commons Attribution 4.0 International license.

10.1128/mBio.00190-19.2 FIG S2 Phenotypic characterization of MFT mutants. (A) Growth of M. smegmatis wild-type (WT) and mutant strains in glycerol (0.2% [vol/vol]). (B and C) Heat map of the enumerated viable bacterial counts during the course of incubation in medium containing (B) cholesterol:EtOH and cholesterol:DMSO as well as (C) the respective solvent control. Each individual value in a matrix represents the total CFU level per milliliter (mean) that was determined from four independent experiments performed in duplicate. (D to G) Bacterial liquid culture in (D) glycerol (0.2% [vol/vol]), (E) propionate (0.5% [wt/vol]), (F) acetate (0.2% [wt/vol]), and (G) acetate (0.5% [wt/vol]) with and without ethanol (1% [vol/vol]). Download FIG S2, TIF file, 1.3 MB. Copyright © 2019 Krishnamoorthy et al. 2019 Krishnamoorthy et al. This content is distributed under the terms of the Creative Commons Attribution 4.0 International license.

10.1128/mBio.00190-19.7 TABLE S1 Strains, plasmids, and PCR primers used in the study. Download Table S1, DOCX file, 0.03 MB. Copyright © 2019 Krishnamoorthy et al. 2019 Krishnamoorthy et al. This content is distributed under the terms of the Creative Commons Attribution 4.0 International license.

Consequently, we assessed whether ethanol would also affect strain Δ*mftC* growth in the presence of another carbon source such as propionate, acetate, or glycerol. Supplementation of ethanol had no effect on strain Δ*mftC* growth in propionate (0.5% [wt/vol]) or glycerol (0.2% [vol/vol]) but severely delayed strain Δ*mftC* growth in acetate (0.5% [wt/vol]) in comparison with the growth of the wild-type strain (Fig. S2D to G). Thus, we conclude that the effect of ethanol on strain Δ*mftC* growth depends on the carbon source and that MFT and ethanol metabolism in M. smegmatis are linked.

### The MFT gene cluster is required for ethanol assimilation in M. smegmatis.

**FIG 3:**
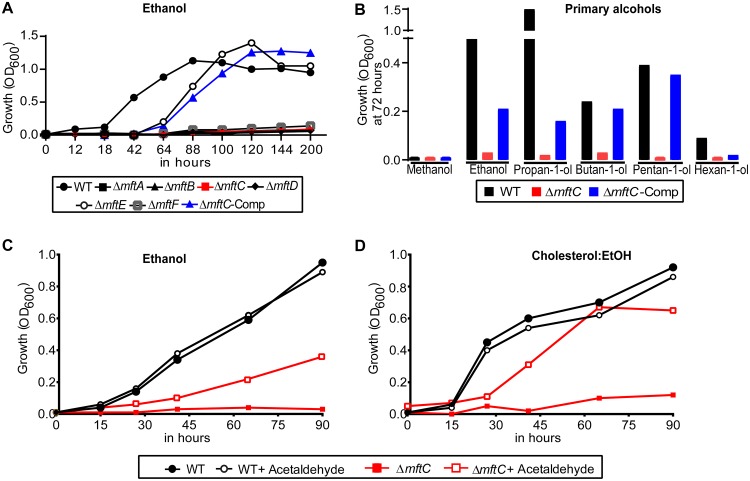
*mft* genes are essential for M. smegmatis growth and metabolism in ethanol. (A) Growth of the *mft* mutants, unlike that of the wild type (WT), was impaired in medium containing ethanol (1% [vol/vol]) as the sole growth substrate. (B) Disruption of *mftC* eliminated the growth of M. smegmatis in other primary alcohols. Data presented in this panel were chosen to represent a time point (at 72 h) of the complete growth assay presented in Fig. S3. (C and D) Acetaldehyde supplementation (0.001% [vol/vol]) partially restored the growth of strain Δ*mftC* in medium containing (C) ethanol and (D) cholesterol:EtOH. Data are representative of results from two independent experiments.

Similar to M. marinum (25), M. smegmatis can utilize ethanol as the sole growth substrate (Fig. 3A; see also Fig. S2C). To directly establish the role of MFT in ethanol assimilation, the growth rates of *mft* mutants and the wild-type strain in ethanol were compared. Strikingly, the *mft* mutants did not grow in ethanol (except for strain Δ*mftE*, which presented delayed growth), whereas the wild-type strain as well as the Δ*mftC*-Comp strains did grow in ethanol. In summary, the function of each *mft* gene affects ethanol assimilation.

10.1128/mBio.00190-19.3 FIG S3 Effect of ethanol on growth of M. smegmatis strains. (A and B) Concentration-dependent growth of (A) the wild-type (WT) strain and (B) the Δ*mftC* mutant in ethanol. Data are representative of results from three independent experiments. (C) Bacterial viability after 2 h of treatment with cell wall-damaging agents (0.015% SDS or 2.5 mg/ml lysozyme) at 37°C under ethanol (1% [vol/vol])-replete conditions. Data represent means ± standard deviations of CFU from three independent experiments. (D to I) Growth of bacterial strains in medium containing (D) no carbon source, (E) methanol (0.5% [vol/vol]; catalog no. 1060092500, Merck), (F) propan-1-ol (0.1%; catalog no. 82090, Fluka), (G) butan-1-ol (0.1%; catalog no. 281549-100ML, Sigma), (H) pentan-1-ol (0.01%; catalog no. 76929-250ML, Sigma), and (I) hexan-1-ol (0.1%; catalog no. 471402-100ML, Sigma). Download FIG S3, TIF file, 1.7 MB. Copyright © 2019 Krishnamoorthy et al. 2019 Krishnamoorthy et al. This content is distributed under the terms of the Creative Commons Attribution 4.0 International license.

The inability of strain Δ*mftC* to grow in ethanol could be attributed to exacerbated toxicity, because high concentrations of ethanol disrupt cell wall and membrane integrity (26, 27). However, ethanol toxicity was not observed as a growth-limiting factor in our study. The growth of strain Δ*mftC* remained unrestored even at the lowest concentration of ethanol tested (Fig. S3A and B), and there was no ethanol-dependent potentiation with respect to the effect of cell wall-damaging agents such as sodium dodecyl sulfate (SDS) and lysozyme (Fig. S3C).

To interrogate the functionality of MFT in catabolism of related substrates, the growth rates of the wild-type strain and Δ*mftC* mutant in other primary alcohols were compared. M. smegmatis grew in all primary alcohols examined (C2 to C6), except in methanol, but with different growth rates under the conditions tested. In contrast, disruption of *mftC* eliminated growth of M. smegmatis (Fig. 3B; see also Fig. S3D to I), thereby strongly establishing the essential role of MFT in primary alcohol assimilation.

### MFT is associated with ethanol oxidation in M. smegmatis.

PQQ-dependent ethanol assimilation involves alcohol and aldehyde dehydrogenases to oxidize ethanol to acetic acid, a redox reaction, with acetaldehyde as a reaction intermediate (14–18). Given the similarities between MFT and PQQ (3)—and the inability of the respective biosynthetic mutants to grow in ethanol (17)—it has been proposed that MFT acts as a redox cofactor to enable the function of alcohol dehydrogenase(s) (3, 13). Accordingly, we assessed whether supplementation of acetaldehyde would rescue strain Δ*mftC* growth in ethanol (probably by compensating for inactive MFT-dependent alcohol dehydrogenase). Indeed, acetaldehyde (0.001% [vol/vol]) supplementation partially restored the growth of strain Δ*mftC* in ethanol (Fig. 3C) as well as in cholesterol:EtOH (Fig. 3D). Neither the mutant nor the wild-type strain grew in medium containing only acetaldehyde (data not shown). Intriguingly, a similar acetaldehyde-dependent growth correction has been reported to eliminate an ethanol-induced lag phase of Saccharomyces cerevisiae via NAD^+^ replenishment by NADH oxidation (28–31). Therefore, disruption of *mft* genes probably causes redox imbalance due to dysregulation/dysfunction of MFT-associated alcohol dehydrogenase in M. smegmatis.

### Ethanol alters the expression of genes associated with respiration and redox homeostasis in M. smegmatis.

**FIG 4:**
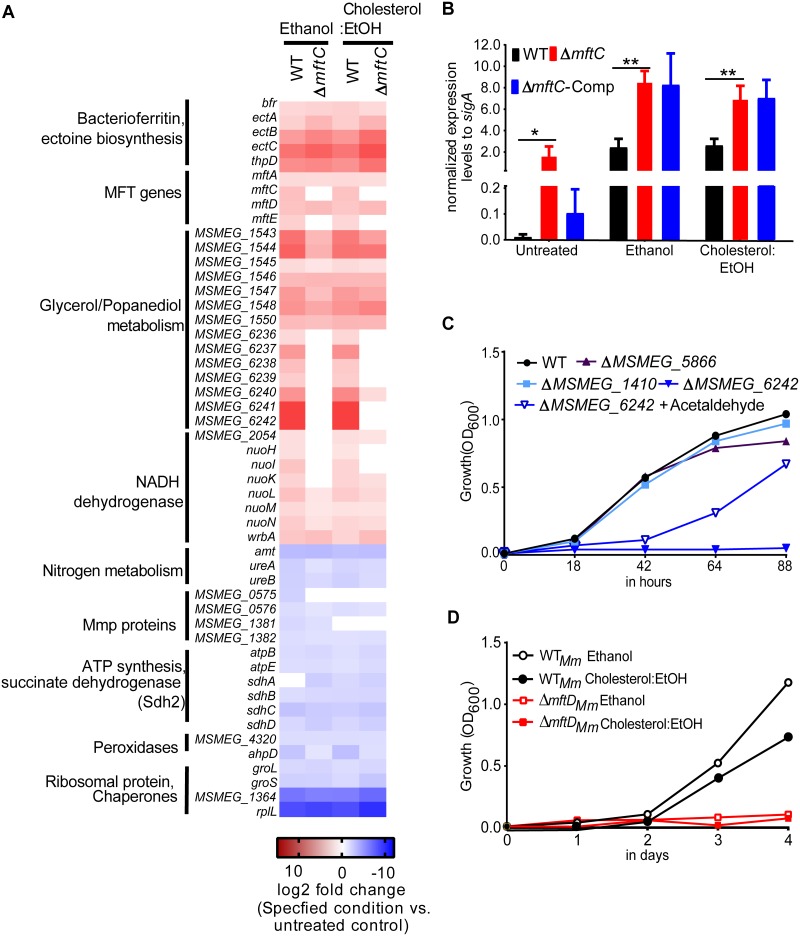
Comparative features of MFT-associated ethanol metabolism in M. smegmatis and M. marinum. An ethanol-induced transcriptomic signature reveals the possible function of MFT and its dependent alcohol dehydrogenase. (A) Transcriptional profile of Δ*mftC* and wild-type (WT) cells treated with ethanol and cholesterol:EtOH for 4 h. The relative expression levels of the differentially expressed genes were compared between conditions or bacterial strains. A gene cluster (*MSMEG_6236* to *MSMEG_6242*) was upregulated only in the WT strain compared with mutants treated with ethanol or cholesterol:EtOH. Intriguingly, the basal expression levels of these genes were significantly higher in untreated mutants (Table S2). (B) qRT-PCR-based quantification of *MSMEG_6242* transcript levels in bacterial strains treated with the specified agents. Expression levels to normalized *sigA* (means ± standard deviations) were calculated from data from three independent experiments performed in triplicates. An unpaired *t* test with Welch's correction was used to determine statistical significance. **, *P < *0.01; *, *P < *0.05. (C) Functional characterization of putative MFT-associated nicotinoproteins. Growth of strain Δ*MSMEG_6242* in ethanol was impaired, while strain Δ*MSMEG_1410* and strain Δ*MSMEG_5866* exhibited WT-like growth. (D) Growth of M. marinum wild type (WT*_Mm_*) and mutant Δ*mftD_Mm_* in ethanol and cholesterol:EtOH. Consistent with the obtained M. smegmatis results, the growth of strain Δ*mftD_Mm_* was impaired. A representative growth curve from three independent experiments is presented.

Microarray-based global gene expression analysis was performed to identify ethanol-inducible genes and to determine the cause of the growth retardation of strain Δ*mftC* in ethanol and cholesterol:EtOH (Fig. 4A; see also Table S2). Following ethanol treatment, genes associated with M. smegmatis respiration (type I NADH:menaquinone oxidoreductases [*nuo* gene cluster]), acetate metabolism, ectoine, B_12_-dependent glycerol dehydrogenase, and propane-diol metabolism, as well as genes encoding several oxidoreductases, were upregulated in both the wild-type strain and Δ*mftC* mutant. In contrast, genes associated with ATP synthase subunits (*atpB* and *atpE*) and Sdh2 (*sdhA*, *sdhB*, *sdhC*, and *sdhD*), genes associated with nitrogen metabolism (ureases and ammonium transporter), and genes *rplL* and *MSMEG_1364* (50S ribosomal protein) were markedly downregulated in both strains after ethanol treatment. In particular, the gene expression profiles of the ethanol-treated and cholesterol:EtOH-treated strains were similar, suggesting that ethanol is preferred in the hierarchy to induce specific transcriptional responses.

10.1128/mBio.00190-19.8 TABLE S2 Transcriptional responses analyzed in this study. Download Table S2, XLSX file, 0.2 MB. Copyright © 2019 Krishnamoorthy et al. 2019 Krishnamoorthy et al. This content is distributed under the terms of the Creative Commons Attribution 4.0 International license.

With regard to the ethanol-induced transcriptional response, *whiB3* (*MSMEG_1831*) and *MSMEG_3644* (MerR family) were upregulated in strain Δ*mftC* in comparison with the wild-type strain. Expression of *MSMEG_6242*, annotated as a glycerol/alcohol dehydrogenase, resulted in higher levels of accumulation in the wild-type strain than in the mutant strains during ethanol treatment (Fig. 4A; see also Table S2). Assessment of this transcriptional modulation by quantitative reverse transcription-PCR (qRT-PCR)-based gene expression analysis revealed that the level of expression of *MSMEG_6242* was significantly higher in strain Δ*mftC*—both untreated and treated—than in the wild-type strain (Fig. 4B). Taking the data together, transcriptional profiling indicated that ethanol affects respiration and redox regulation and that the putative alcohol dehydrogenase MSMEG_6242, requires MFT for its function.

### MSMEG_6242 is a putative MFT-associated alcohol dehydrogenase.

To validate the role of *MSMEG_6242* in ethanol assimilation in M. smegmatis, a deletion mutant was constructed (Fig. S1) and phenotyped. Strikingly, the Δ*MSMEG_6242* mutant failed to grow in ethanol (Fig. 4C) as well as in cholesterol:EtOH (Fig. S4). However, this growth impairment was corrected, as in the case of strain Δ*mftC*, by the supplementation of acetaldehyde, thus confirming the role of *MSMEG_6242* in ethanol assimilation.

10.1128/mBio.00190-19.4 FIG S4 Growth phenotype of different M. smegmatis mutants in cholesterol:EtOH. (A) In addition to strain Δ*MSMEG_6242*, the other mutant strains (strains Δ*MSMEG_1410* and Δ*MSMEG_5866*) grew in cholesterol:EtOH. Supplementation with 0.001% acetaldehyde (catalog no. 402788-100ML, Sigma) partly rescued the growth of strain Δ*MSMEG_6242* in cholesterol:EtOH. Data are representative of results from three independent experiments. Download FIG S4, TIF file, 1.4 MB. Copyright © 2019 Krishnamoorthy et al. 2019 Krishnamoorthy et al. This content is distributed under the terms of the Creative Commons Attribution 4.0 International license.

In addition to *MSMEG_6242*, other oxidoreductase/dehydrogenase genes (*MSMEG_1410*, *MSMEG_2687*, and *MSMEG_5866*) have been predicted to be probable MFT-associated nicotinoproteins, given their co-occurrence with *mft* genes in many bacterial genomes (3, 13). The role of these dehydrogenases in ethanol assimilation was analyzed in a genetic approach. While repeated attempts to isolate a viable *MSMEG_2687* mutant strain were unsuccessful, mutant strains lacking *MSMEG_5866* or *MSMEG_1410* were readily isolated (Fig. S1). Both of those mutant strains—unlike the Δ*MSMEG_6242* and Δ*mftC* mutants—showed wild-type-like growth in ethanol and cholesterol:EtOH (Fig. 4C; see also Fig. S4), suggesting their dispensability or redundancy for ethanol assimilation. Given that the mutant’s growth phenotype can be used as a criterion to establish functional association—along with prior bioinformatics predictions (3)—*MSMEG_6242* can be regarded as a putative MFT-dependent alcohol dehydrogenase gene required for M. smegmatis growth in ethanol.

Note that no direct homolog of *MSMEG_6242* is present in either M. marinum and M. tuberculosis; however, only the latter grows poorly in ethanol (22, 25), suggesting species-specific variations in MFT-associated growth and metabolism in ethanol. To interrogate the relevance of MFT for the growth of M. marinum in the presence of ethanol and cholesterol:EtOH, we disrupted *mftD* as a representative of the *mft* genes (Table S1). Strikingly, the growth of M. marinum Δ*mftD* (Δ*mftD_Mm_*) was impaired in minimal medium containing ethanol (0.5% [vol/vol]) or cholesterol:EtOH, thus emphasizing the significance of MFT in ethanol assimilation as well as establishing the functional existence of an MFT-associated but nonorthologous alternative for *MSMEG_6242* in M. marinum (Fig. 4D).

### Ethanol limits growth of *mftC* mutant of M. tuberculosis in cholesterol.

**FIG 5:**
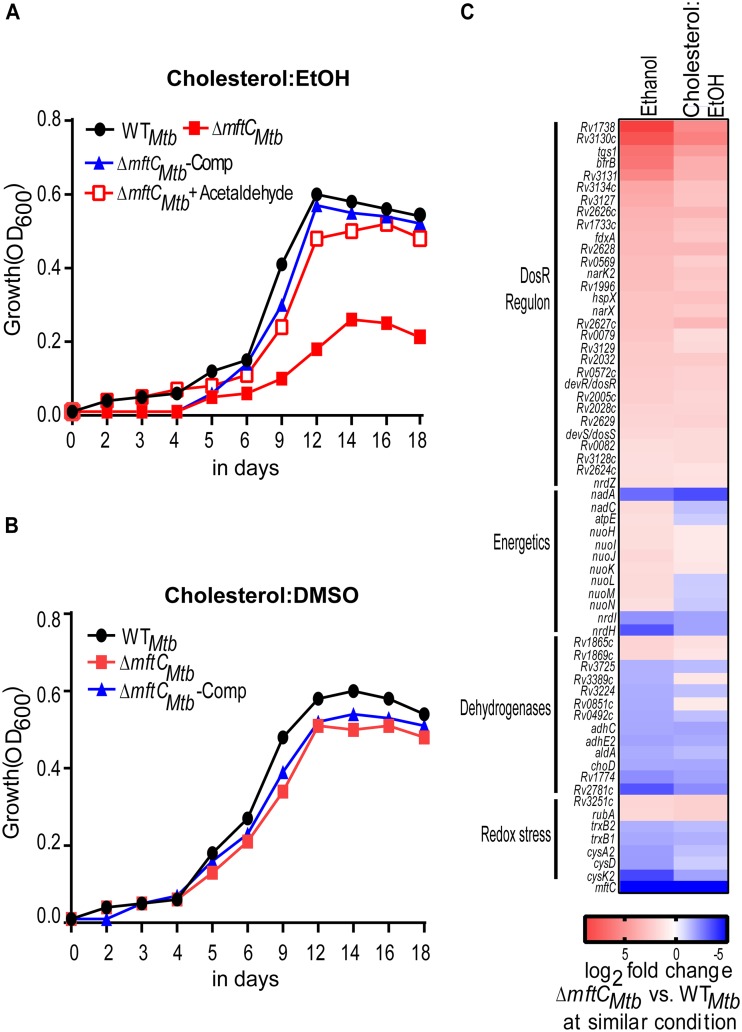
Disruption of *mftC* impacts M. tuberculosis growth in cholesterol:EtOH. (A and B) Growth of strain Δ*mftC_Mtb_* and wild-type M. tuberculosis (WT*_Mtb_*) in (A) cholesterol:EtOH and (B) cholesterol:DMSO. A representative growth curve from two independent experiments is presented. (C) The transcriptional response of strain Δ*mftC_Mtb_* treated with cholesterol:EtOH largely mimics the ethanol-treated condition. DosR-regulated genes were upregulated, while genes associated with respiration and redox homeostasis were differentially regulated in strain Δ*mftC_Mtb_*.

Unlike M. smegmatis or M. marinum, M. tuberculosis has been found to grow poorly in ethanol (22). To validate the function of MFT in M. tuberculosis, an in-frame deletion mutant lacking *mftC*, here referred to as *ΔmftC_Mtb_*, was constructed (Fig. S1). As expected, strain Δ*mftC_Mtb_* exhibited a partial growth defect in medium containing cholesterol as the sole carbon source, and a genetically complemented derivative, strain Δ*mftC_Mtb_*-Comp, showed restored growth in cholesterol:EtOH (Fig. 5A). Notably, the growth of strain Δ*mftC_Mtb_* was not severely retarded in cholesterol:DMSO (Fig. 5B). Because the M. tuberculosis wild-type strain presented poor growth in ethanol, growth deficiency, if any, in the mutant was undetectable (Fig. S5A). However, similar to the results seen with M. smegmatis, acetaldehyde supplementation restored the growth of strain Δ*mftC_Mtb_* in cholesterol:EtOH to a level comparable to that seen with the wild-type strain (Fig. 5A). Moreover, growth of strain Δ*mftC_Mtb_* was unaffected in glycerol, or in acetate, but was partly affected in propionate under conditions of ethanol repletion (Fig. S5B to D). These results suggest that the function of *mftC* is conserved between M. smegmatis and M. tuberculosis, regardless of their differential ability to grow in ethanol.

10.1128/mBio.00190-19.5 FIG S5 Effect of ethanol on the growth of M. tuberculosis strains. (A) Poor growth of the M. tuberculosis wild type (WT) and strain Δ*mftC_Mtb_* in ethanol (0.2% [vol/vol]). (B to D) Bacterial growth in (B) glycerol (0.2% [vol/vol]), (C) acetate (0.1% [wt/vol]), (D) propionate (0.1% [wt/vol]) with and without ethanol (1% [vol/vol]). Data are representative of results from two independent experiments. Download FIG S5, TIF file, 1.9 MB. Copyright © 2019 Krishnamoorthy et al. 2019 Krishnamoorthy et al. This content is distributed under the terms of the Creative Commons Attribution 4.0 International license.

### Transcriptional response of strain Δ*mftC_Mtb_* upon ethanol and cholesterol:EtOH treatment.

Microarray-based global gene expression profiling was performed to investigate the impact of *mftC* deletion on the ethanol-induced M. tuberculosis transcriptional response (Fig. 5C; see also Table S2). Comparisons of the responses of wild-type M. tuberculosis and strain Δ*mftC_Mtb_* revealed that most of the DosR regulon genes were significantly upregulated in the mutant, indicating a dysfunctional respiratory or redox imbalance state of the ethanol-treated mutant. In contrast, genes involved in cell wall biogenesis (*kasA* [−1.6-fold]) and *kasB* [−1.8-fold]) as well as genes encoding iron-dependent enzymes (*cysK2* [−3.6-fold], *furA* [−2.6-fold], and *lytB2* [−3.5-fold]) were markedly downregulated in the mutant.

To examine the effect of *mftC* deletion on cholesterol utilization by M. tuberculosis, the transcriptional responses of strain Δ*mftC_Mtb_* treated with cholesterol:EtOH were compared with those of the untreated control. Most notably, the genes required for cholesterol catabolism—from sterol degradation (*kstR2*, *fadE30*, *echA20*, *kshA*, *hsaA*, and *hsaD*) to propionate utilization (*Rv1130*, *Rv1131*, and *icl1*)—were upregulated in cholesterol:EtOH-treated strain Δ*mftC_Mtb_*. In contrast, approximately 2-fold reductions in expression of genes involved in acetate metabolism (*pdhA*, *pdhB*, and *pdhC*) and in respiration (*nadA*, *nadB*, and *nadC* and *cydA*) were observed in strain Δ*mftC_Mtb_*. Moreover, the levels of gene expression of ethanol-treated and cholesterol:EtOH-treated M. tuberculosis largely overlapped (Table S2 and S3).

10.1128/mBio.00190-19.9 TABLE S3 Gene expression values from cholesterol:EtOH-treated strain Δ*mftC_Mtb_* relative to untreated cells. Download Table S3, DOC file, 0.1 MB. Copyright © 2019 Krishnamoorthy et al. 2019 Krishnamoorthy et al. This content is distributed under the terms of the Creative Commons Attribution 4.0 International license.

### Ethanol treatment does not impact redox and bioenergetics parameters in the *mftC* mutant.

**FIG 6:**
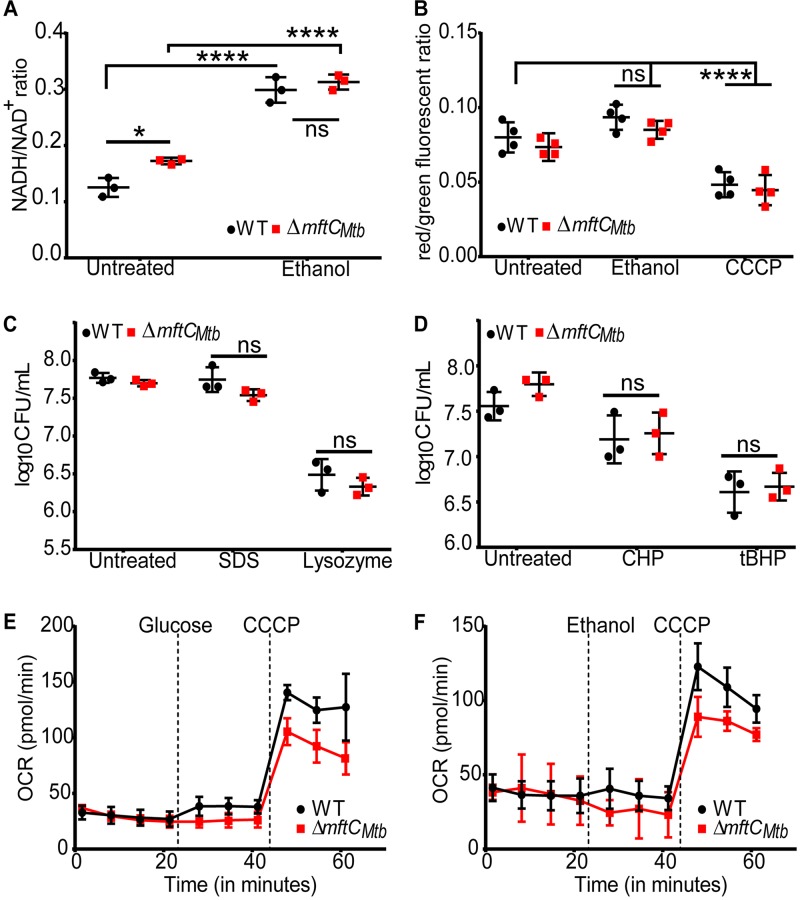
Disruption of *mftC* does not impact ethanol-induced changes in redox status and basal respiratory capacity. (A) Ethanol treatment of wild-type M. tuberculosis (WT) and strain Δ*mftC_Mtb_* for 24 h altered the NADH/NAD^+^ ratio but was independent of *mftC* function. (B) Membrane potential was unaltered in the presence of ethanol (1% [vol/vol]). Cells treated with 20 μM carbonyl cyanide *m*-chlorophenyl hydrazine (CCCP) for 30 min were used as a positive control. ****, *P* < 0.001 (two-way ANOVA/Tukey’s multiple-comparison test). (C and D) Notably, strain Δ*mftC_Mtb_* was not increasingly susceptible to (C) cell wall-damaging agents (0.015% SDS or 2.5 mg/ml lysozyme) or (D) oxidative-stress-inducing agents (1 mM CHP or 1 mM tBHP) in the presence of ethanol (1% [vol/vol]). ns, not significant (one-way ANOVA/Tukey’s multiple-comparison test on log-transformed data). (E and F) No difference in OCR, measured using an Agilent Seahorse XFp analyzer, was noted. Time points for the addition of compounds are indicated by vertical dotted lines. The final well concentrations were 1 mM (glucose), 1% (ethanol), and 5 μM (CCCP). The OCR data points are representative of the average OCR during 4 min of continuous measurement in the transient microchamber. Standard deviations were calculated from the OCR measurements taken from three replicate wells by the use of Wave Desktop 2.4.0 software. Pooled data from three independent experiments showed no statistically significant differences (two-way ANOVA/Tukey’s multiple-comparison test) between the conditions.

Transcriptional analysis indicated that ethanol treatment affects the respiration and redox status of the *mftC* mutant. We speculated that the cytosolic NADH/NAD^+^ ratio—which can reliably indicate redox status—is altered upon ethanol treatment. Indeed, the NADH/NAD^+^ ratio was increased in the ethanol-treated wild-type and *ΔmftC_Mtb_* strains (Fig. 6A) in comparison with the untreated culture. Intriguingly, there was no mutant-specific defect in recycling of NADH and NAD^+^. In addition, there were no differences in membrane potential (Fig. 6B) and in susceptibility to cell wall-damaging agents (Fig. 6C) in both the wild-type and *ΔmftC_Mtb_* strains following 24 h of ethanol treatment (Fig. 6B and C). Furthermore, ethanol treatment did not enhance the susceptibility of the wild-type and mutant strains to agents inducing redox stress (1 mM cumene hydroperoxide [CHP] or *tert*-butyl hydroperoxide [tBHP]), suggesting that MFT is not directly associated with cellular oxidative stress responses (Fig. 6D). Likewise, there were no differences between the wild-type and *ΔmftC_Mtb_* strains with respect to the oxygen consumption rate (OCR), as measured by the use of an extracellular flux analyzer (Fig. 6E and F). Most notably, similar findings were also obtained with M. smegmatis strains (Fig. S6). Although transcriptional evidence suggested a role for MFT in respiration/redox regulation, none of these results were confirmatory. Thus, MFT may be involved in a noncanonical redox mechanism in mycobacteria.

10.1128/mBio.00190-19.6 FIG S6 Distinct role of MFT in M. smegmatis redox regulation. (A) Four hours of ethanol treatment altered the NADH/NAD^+^ ratio of both the wild-type (WT) and Δ*mftC* strains. (B) However, there was no difference in membrane potential. *, *P* < 0.05; ****, *P* < 0.001; ns, not significant (two-way analysis of variance [ANOVA]/Tukey’s multiple-comparison test). (C) Strain Δ*mftC* was not increasingly susceptible to oxidative-stress-inducing-agent (cumene hydroperoxide) treatment—for 30 min or 120 min—in the presence of 1% ethanol (vol/vol). Data represent means ± standard deviations (SD) of results from two independent experiments. One-way ANOVA/Tukey’s multiple-comparison test was performed on log-transformed data. (D and E) No difference in the OCR, as measured using an Agilent Seahorse XFe96 analyzer, was observed. Time points for the addition of assay medium, ethanol, or CCCP are indicated by vertical dotted lines. The OCR data points are representative of the average OCR during 4 min of continuous measurement in the transient microchamber. Standard deviations were calculated from the OCR measurements taken from four replicate wells by the use of Wave Desktop 2.4.0 software. Pooled data from three independent experiments showed no statistically significant differences (two-way ANOVA/Tukey’s multiple-comparison test) between the conditions. Download FIG S6, TIF file, 1.7 MB. Copyright © 2019 Krishnamoorthy et al. 2019 Krishnamoorthy et al. This content is distributed under the terms of the Creative Commons Attribution 4.0 International license.

## DISCUSSION

Our results establish the significance of MFT in mycobacterial metabolism of primary alcohols complementing a range of biological functions documented for RiPPs. So far, PQQ-dependent ethanol oxidation by acetic acid bacteria, which is exploited for the industrial production of vinegar, has been well characterized (14–18). Intriguingly, the M. smegmatis genome (but not the M. tuberculosis or M. marinum genomes) comprises genes putatively involved in biosynthesis of PQQ and its dependent alcohol dehydrogenases (*MSMEG_3721* to *MSMEG_3727*) (3). However, the established genetic requirement of MFT for the growth of M. smegmatis in ethanol excludes the possibility of overlapping functions of PQQ-dependent reactions under the *in vitro* conditions examined in the present study.

While MFT biosynthesis genes are conserved, the numbers of predicted MFT-associated nicotinoproteins encoded in mycobacterial genomes differ greatly and could impact the ethanol assimilatory capacity—complete or partial—of each mycobacterial species. For instance, the lack of a homolog of *MSMEG_6242* in M. tuberculosis could account for its poor growth in ethanol. Yet M. marinum, lacking a direct homolog of *MSMEG_6242*, was found to exhibit sustained growth in ethanol, probably owing to the functionality of other MFT-associated dehydrogenases. Moreover, the proposition of a functional relationship between MFT and *MSMEG_6242* is solely based on the mutant’s shared phenotypes and on prior bioinformatics prediction (3) and thus requires further biochemical confirmation. Nevertheless, the established role of MSMEG_6242 in ethanol assimilation adds to the functional diversity of this enzyme in methylotrophy (32, 33) and in glycerol metabolism and as a target for posttranscriptional/posttranslational regulation and *S*-mycothiolation (34–36). Therefore, MSMEG_6242, and probably also MFT, is likely to function at the crossroads of regulation, metabolism, and growth in M. smegmatis. In contrast, ethanol metabolism—and related MFT function—appears to be irrelevant for M. tuberculosis, perhaps because the status of availability of these volatile compounds with hydroxy groups during infection is ambiguous. Nonetheless, ethanol, as a poor growth substrate for M. tuberculosis, has been demonstrated to alter the cellular state, with NADH accumulation corresponding to the increase in expression of DosR regulon genes (37), mimicking ascorbate treatment (38) and hypoxia adaptation (39). Accordingly, an inability to support active growth is not a strong criterion to predict the impact of a substrate on cellular respiration. Therefore, it is tempting to speculate that a similar poor growth substrate, particularly under nutrient/oxygen-deprived conditions (40, 41), could contribute to respiratory flexibility and, in turn, prolong the survival of M. tuberculosis.

Mycobacterial respiration under hostile conditions requires utilization of different electron donors and acceptors, the diversity of which is not completely known (19, 41). To this end, the proposed function of MFT as an electron carrier involved in redox exchange with its nicotinoprotein partners gains significance (3). The present study demonstrated that ethanol-induced transcriptional signatures exhibit strong associations between MFT function and redox homeostasis/respiration. Notably, loss of function of *mftC* resulted in multifold increases in expression of the DosR regulon genes in ethanol-treated M. tuberculosis. The DosR regulon expression is triggered by the redox sensor DosS in response to a reduced menaquinone pool of the respiratory chain (38, 42). Furthermore, MftC contains at least two iron-sulfur clusters with an intrinsic ability to regulate the redox state of bacterial cells (3, 5, 6, 43). This notion is further supported by the identification of *mft* genes as part of a transcriptional network, which are differentially regulated in response to (or in compensation for) the loss of function of *whiB4*, which recognizes the intracellular redox signals in M. tuberculosis (44). Nonetheless, we noted that the M. tuberculosis strain lacking *mftC* function was neither hypersusceptible to redox stressors nor impaired in recycling nicotinamide cofactors in comparison with the wild-type strain in the presence of ethanol. One possible reason for this lack of differences in NADH/NAD^+^ recycling could be that the MFT-associated nicotinoprotein probably does not rely on cofactor exchange but instead uses MFT as an external redox partner and a nonexchangeable NAD for multiple turnover, as suggested earlier (3, 13). Alternatively, the functions of MFT are likely analogous to those of PQQ, which acts as a redox couple (PQQ/PQQH_2_) during alcohol oxidation in quinoprotein dehydrogenase (14, 18, 45–47). This PQQ-dependent enzyme is known to donate electrons to *c*-type cytochromes or directly to ubiquinone instead of using NAD^+^ or NADP^+^ as an electron acceptor (48). Therefore, understanding the nature of MFT-associated redox chemistry (with its nicotinoprotein partners) and of its impact on mycobacterial cellular metabolism is crucial and the subject of ongoing investigation.

Intriguingly, we found that disruption of *mft* genes profoundly impaired the ability of mycobacterial species to assimilate cholesterol in the presence of ethanol. Griffin et al. (20) pioneered the identification of essential genes for cholesterol catabolism by comparing mutant pools grown under glycerol:EtOH and cholesterol:EtOH conditions. Here we found that, remarkably, ethanol selectively compromised the ability of strain Δ*mftC* to assimilate cholesterol, but not glycerol, which likely influenced the essentiality prediction of MFT genes. Similarly, Nazarova et al. (21) demonstrated that disruption of *mft* genes alleviated the growth defect of M. tuberculosis strain Δ*icl1*::*hyg* in the presence of cholesterol:EtOH and a carbohydrate. We propose that, under such conditions, ethanol-derived suboptimal cholesterol catabolism (by partial degradation of the carbon) in strain Δ*mftC* reduces propionyl-coenzyme A (propionyl-CoA) flux and consequently methylcitrate toxicity, enabling the growth of strain Δ*icl1*::*hyg*. Although the effect of ethanol was obvious with additional results obtained from M. smegmatis and M. marinum, the possibility of an impact of other constituents of media on growth cannot be precluded. Nevertheless, in addition to the results seen with cholesterol, the rate of growth of M. smegmatis in acetate was also restricted by ethanol and was reminiscent of catabolite repression. This effect was, however, less pronounced in M. tuberculosis, implying species-specific differences in potential mechanisms of ethanol-induced inhibition of cholesterol metabolism. The mechanisms underlying ethanol-induced growth stasis/impairment are multifactorial, as observed in other bacterial systems: ethanol (i) impairs the regeneration of NADH/NAD^+^ (29–31); (ii) affects the uptake of acetate (16, 49–51); and (iii) suppresses the genes required for acetate assimilation and metabolic turnover of acetyl-CoA (52–54). Although these factors may still be relevant in the case of strain Δ*mftC* growth impairment in acetate or cholesterol:EtOH, our transcriptomic analysis suggests that the extent of ethanol-induced growth impairment is probably owed to compounded and yet species-specific effects of redox imbalance and altered bioenergetics.

Our genetic analysis adds to the expanding knowledge about MFT biosynthesis (3, 5–9) in the context of ethanol metabolism. However, the inferred functional discordance of the data resulting from analyses of the *mftE* gene was unexpected, despite its well-recognized significance in MFT maturation (7–9). Nevertheless, the growth permissiveness of strain Δ*mftE* in ethanol could have been the result of residual functions of an end metabolite(s) lacking an MftE-mediated modification(s) or of other compensatory gene functions. Complementing the ongoing efforts (5–9) in elucidating the significance of *mftE* and other *mft* genes in the structural/functional diversity of MftA-derived metabolites, future studies should directly investigate nonpathogenic M. smegmatis strain as a more appropriate natural host with fully functional associated metabolic pathways.

In conclusion, we identified a previously unknown function of MFT, a peptide-derived cofactor, in ethanol assimilation in mycobacteria. MFT is most likely associated with several other biochemical processes, because it is predicted to act as a redox cofactor for other dehydrogenases whose functions were shown here to be unrelated to ethanol metabolism. Although the role of MFT in M. tuberculosis survival, persistence, and pathogenesis cannot be reliably predicted, the complete loss of MFT biosynthesis in M. leprae (3) and species-specific variations in genes encoding MFT-associated dehydrogenases suggest, at the least, a selective advantage for the retention of MFT in mycobacterial species to inhabit diverse ecological niches.

## MATERIALS AND METHODS

### Bacteria, media, and growth conditions.

Strains used in this study are listed in Table S1 in the supplemental material. Escherichia coli DH5α was propagated in Luria-Bertani (LB) medium at 37°C or on LB agar plates containing no or appropriate antibiotics. Mycobacterial strains were propagated in Middlebrook 7H9 broth (Difco) supplemented with albumin-dextrose-catalase enrichment (Becton, Dickinson), 0.2% glycerol, and 0.05% Tyloxapol at 37°C with agitation/rotation (100 rpm) or on Middlebrook 7H11 agar containing 10% (vol/vol) oleic acid-albumin-dextrose-catalase enrichment (Becton, Dickinson) at 37°C. All other chemicals were from Sigma-Aldrich/Fisher Scientific unless otherwise specified.

Carbon utilization assays were performed using minimal medium as described earlier (20). Cholesterol (0.01%) was solubilized in 0.5% hot ethanol or 1% hot DMSO. Other carbon sources tested were sodium acetate (0.5%), sodium propionate (0.1% or 0.5%), ethanol (0.01% to 1%), methanol (0.5%), propan-1-ol (0.1%), butan-1-ol (0.1%), pentan-1-ol (0.01%), and hexan-1-ol (0.1%). Wherever appropriate, 0.001% acetaldehyde was supplemented. To determine susceptibility to redox stressors, bacterial cells at the log phase of growth were treated with 1 mM CHP or tBHP for 1 h (M. tuberculosis) and 2 h (M. smegmatis) under conditions of ethanol repletion at 37°C. To assess the impact of cell wall-damaging agents, the bacterial cells at the log phase of growth were treated with 0.015% of SDS or with 2.5 mg/ml lysozyme for 2 h at 37°C in the presence of 1% ethanol. The recovered bacterial CFU were enumerated.

### Construction of mutants.

Allelic exchange mutants were constructed by two-step selection using the p2NIL/pGOAL system (55). Bacterial genotyping was done by PCR and Southern blotting. Genetic complementation studies used integration plasmid pMCpAINT (56). The details of PCR oligonucleotides and suicide plasmids are provided in Table S1.

### RNA extraction and microarray analysis.

Bacterial strains were treated with 1% ethanol or cholesterol:EtOH (0.01% cholesterol–1% ethanol) for 4 h (M. smegmatis) or 24 h (M. tuberculosis). RNA was extracted with TRIzol reagent (Invitrogen). Cell lysis was achieved through three cycles of bead beating in a FastPrep-24 instrument (MP) at 4.5 m/s for 30 s. RNA was prepared using a Turbo DNA-free kit (Ambion), and RNA concentrations and quality were determined with a NanoDrop ND-1000 spectrophotometer and an Agilent 2100 Bioanalyzer, respectively. Quantitative PCR (qPCR) was performed with Fast SYBR green Master Mix kit (Applied Biosystems).

Microarray experiments were performed as dual-color hybridizations on custom M. smegmatis 4-by-44K (Agilent-017184/expression) and on custom 8-by-60K M. bovis BCG—M. tuberculosis H37Rv M. tuberculosis V3 (Agilent-035148/multipack) arrays. To compensate for dye-specific effects, dye-reversal color-swap hybridizations were applied. Total RNA labeling and amplification were performed using a Quick Amp gene expression labeling kit (Agilent Technologies) and a 5:1 mixture of FullSpectrum MultiStart T7 primer (BioCat GmbH) with oligo(dT)-T7 primer (Agilent Technologies). After precipitation, purification, and quantification, 0.75-μg volumes of both Cy-labeled cRNAs were fragmented and hybridized to Agilent-035148 or 1.25 μg of each labeled cRNA was used with Agilent-017184. Scanning of microarrays was performed with either 3-μm resolution and 20 bits (8 by 60K) or an extended dynamic range and 5-μm resolution and 16 bits (4 by 44K) using a G2565CA high-resolution laser microarray scanner (Agilent Technologies). Microarray image data were processed using Image Analysis/Feature Extraction G2567AA v. A.11.5.1.1 software and default settings and the GE2_1105_Oct12 extraction protocol. The extracted MAGE-ML files were analyzed with Rosetta Resolver Biosoftware, Build 7.2.2 SP1.31 (Rosetta Biosoftware). Ratio profiles comprising single hybridizations were combined in an error-weighted fashion to create ratio experiments. A 1.5-fold change expression cutoff value for ratio experiments was applied together with dye-swapped ratio profiles, rendering the microarray analysis data highly significant (*P* < 0.01), robust, and reproducible (57). Alternatively, the extracted raw data files (.txt) were analyzed using R and the associated BioConductor limma R package (58).

### Measurement of NAD/NADH levels and membrane potential.

Bacterial cells were subjected to ethanol treatment for 4 h (M. smegmatis) or 24 h (M. tuberculosis). The NAD and NADH concentrations were determined using a FluroNAD/NADH detection kit (Cell Technology). The membrane potential of M. smegmatis strains was determined after incubation with DiCO2 fluorescent dye (3 mM) for 30 min and was further analyzed on a BD fluorescence-activated cell sorter (FACS) LSRII instrument, whereas the membrane potential of M. tuberculosis strains was measured as previously reported (59) with some modifications. Samples were transferred into clear-bottom 96-well microtiter plates and centrifuged at 3,500 × *g* for 10 min. DiOC2 (15 μM) was then added to each well and incubated for 20 min at room temperature, followed by fixation with 4% formaldehyde for an additional 60 min. Subsequently, samples were washed with fresh 7H9 medium to remove extracellular dye. A SpectraMax M5 spectrofluorimeter (Molecular Devices) was used to measure green fluorescence (488-nm wavelength/530-nm wavelength) and shifts to red fluorescence (488-nm wavelength/610-nm wavelength). Membrane potential was determined as the ratio of red fluorescence to green fluorescence. Untreated and carbonyl cyanide *m*-chlorophenyl hydrazine (CCCP)-treated cells were included as controls. Each condition was measured in triplicate, and each experiment was performed at least twice.

### Extracellular flux analysis.

OCR values were measured as described earlier (60), with some modifications, using unbuffered (pH 7.35) minimal medium (M. smegmatis) and Middlebrook 7H9 medium (M. tuberculosis) free from any carbon sources.

For the M. smegmatis experiments, a culture in the mid-log phase of growth (optical density at 600 nm [OD_600_] of 0.8) was starved for 6 h in carbon source-free minimal medium containing 0.01% tyloxapol. Then, 2 × 10^5^ to 4 × 10^5^ cells/well were added to a Cell-Tak (Corning)-coated XF 96 cell culture microplate and were analyzed using an Agilent Seahorse XFe96 analyzer. OCR values were measured for about 21 min before the addition of preloaded ethanol (1%) or of medium through injection ports of the sensor cartridge. Finally, 5 mM CCCP was added to the wells and the measurement was continued for another 21 min.

For the M. tuberculosis experiments, a culture in the log phase of growth (OD_600_ of 0.6 to 0.8) was starved for 18 to 24 h in carbon source-free 7H9 medium containing 0.01% tyloxapol. Subsequently, about 5 × 10^5^ cells/well were added to a Cell-Tak-coated XFp miniplate and analyzed using an Agilent Seahorse XFp analyzer. The assay conditions were similar to those described for M. smegmatis.

### Statistical analysis.

GraphPad Prism 7.03 software was used for statistical analyses. *P* values of less than 0.05 were considered statistically significant.

### Data availability.

Microarray data are available from the NCBI GEO database under accession no. GSE121398.
