# Supplementary figures and images for: Mycofactocin Is Associated with Ethanol Metabolism in Mycobacteria
Source: mBio. 2019 May 21;10(3):e00190-19. doi: 10.1128/mBio.00190-19 (PMC6529628; doi:10.1128/mBio.00190-19)

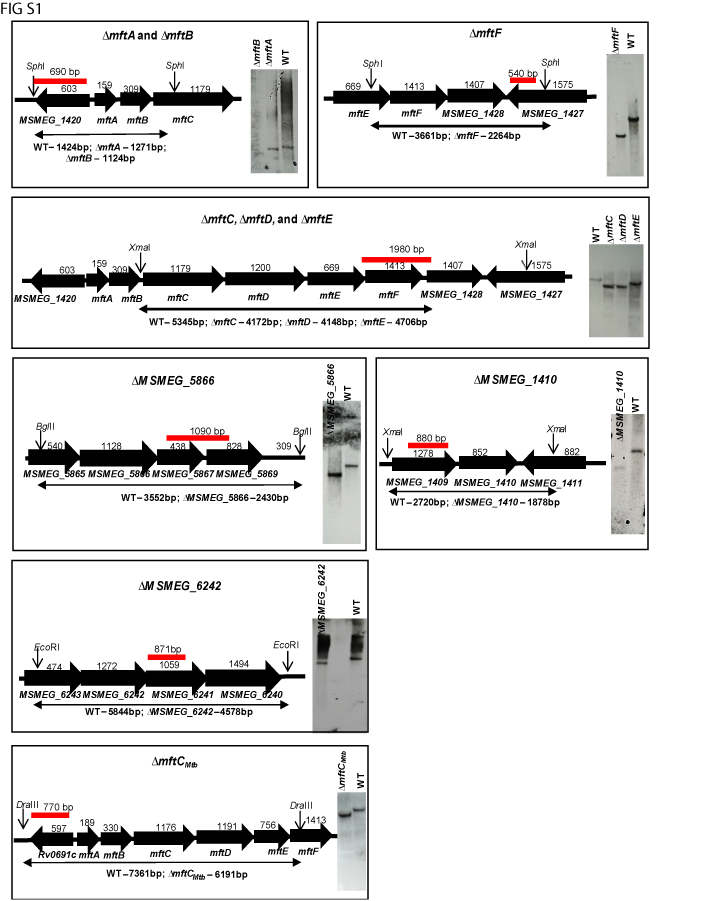

Supplement: FIG S1 [file mBio.00190-19-sf001.tif]

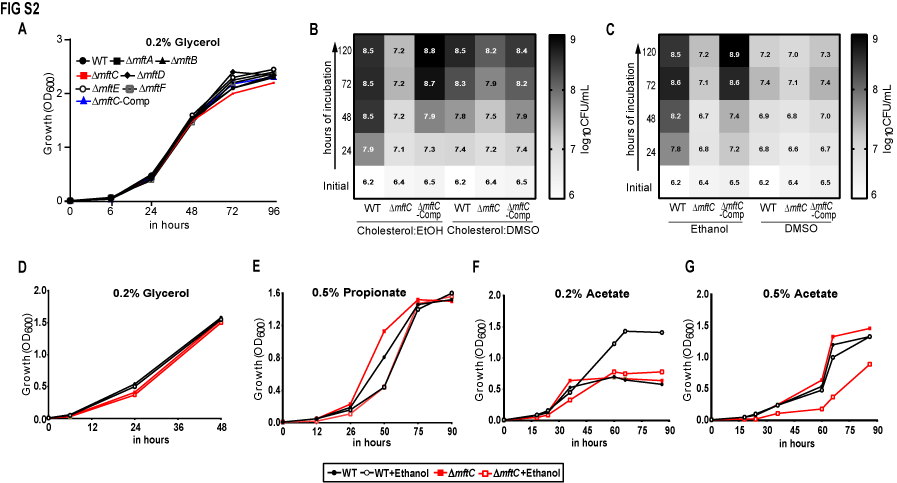

Supplement: FIG S2 [file mBio.00190-19-sf002.tif]

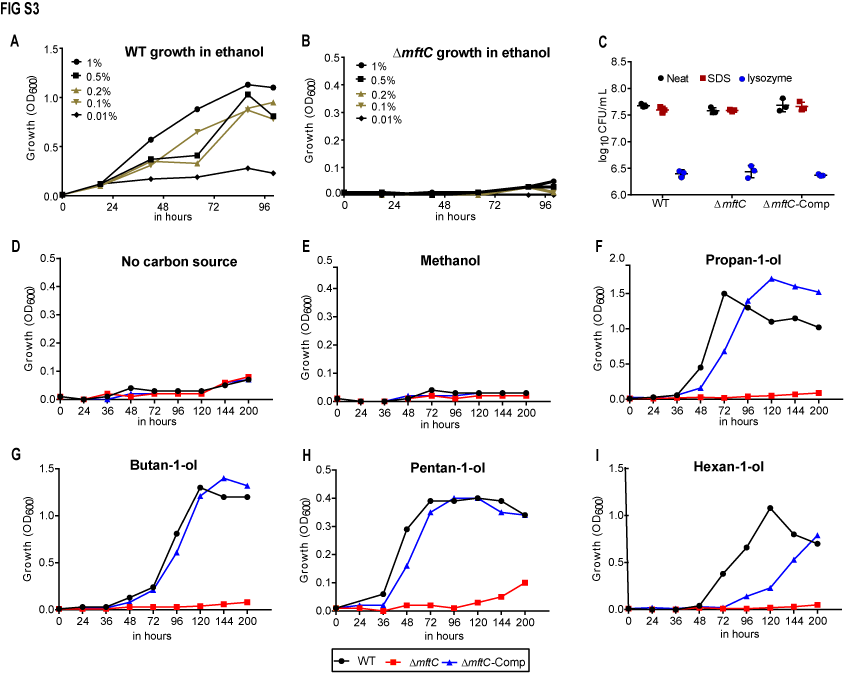

Supplement: FIG S3 [file mBio.00190-19-sf003.tif]

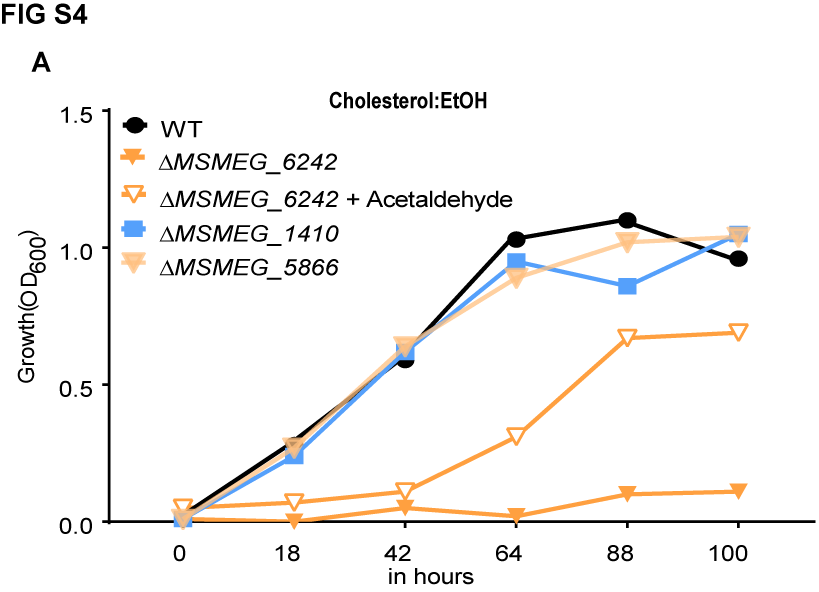

Supplement: FIG S4 [file mBio.00190-19-sf004.tif]

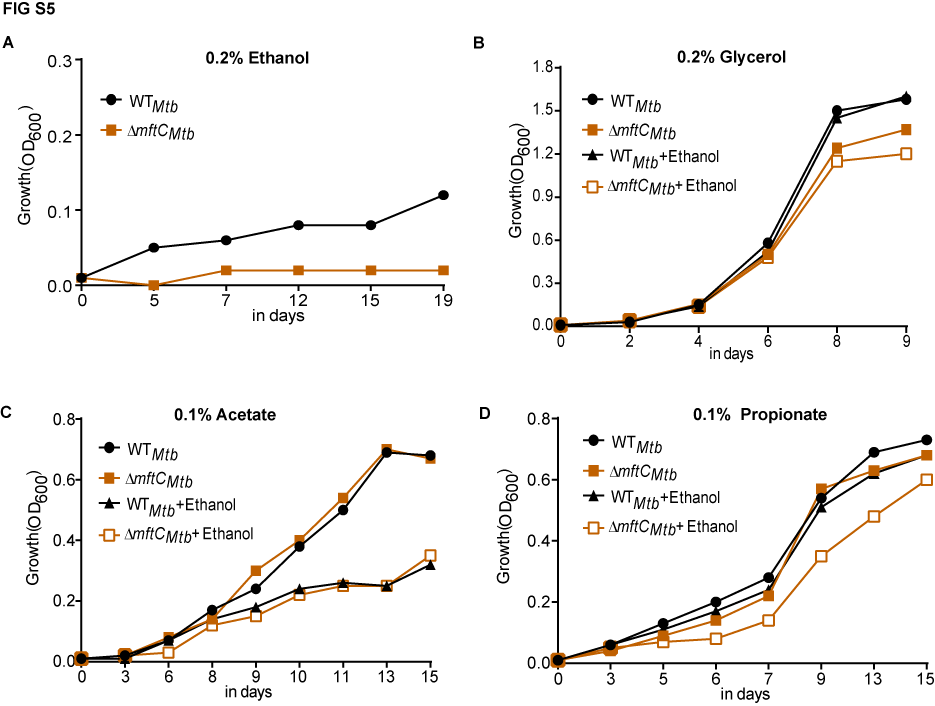

Supplement: FIG S5 [file mBio.00190-19-sf005.tif]

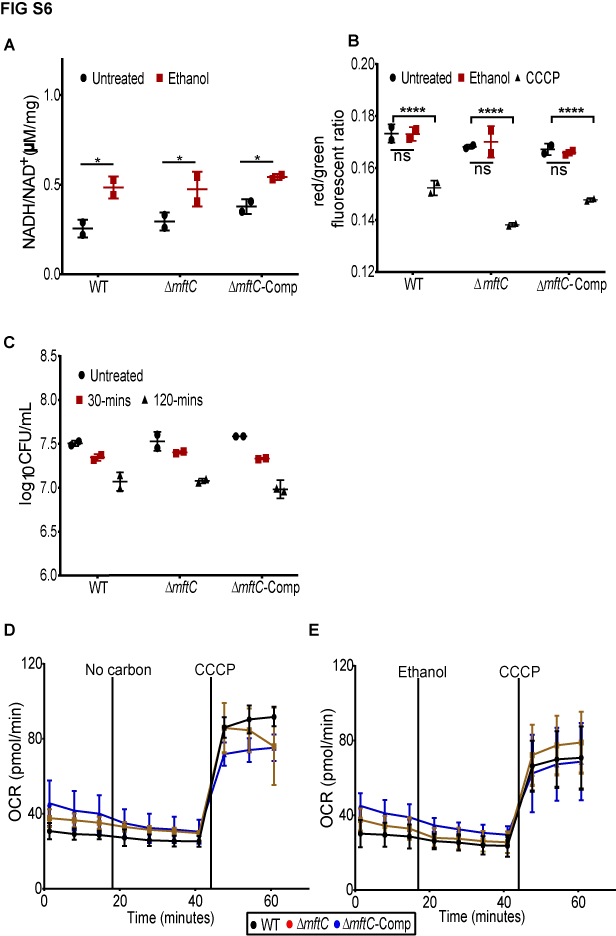

Supplement: FIG S6 [file mBio.00190-19-sf006.tif]
