# Supplementary material for: Mycofactocin Is Associated with Ethanol Metabolism in Mycobacteria
Source: mBio. 2019 May 21;10(3):e00190-19. doi: 10.1128/mBio.00190-19 (PMC6529628; doi:10.1128/mBio.00190-19)
Supplement: TABLE S1 [file mBio.00190-19-st001.docx]

| Gopinath Krishnamoorthy, Peggy Kaiser, Laura Lozza, Karin Hahnke, Hans-Joachim Mollenkopf, Stefan H. E. Kaufmann. Mycofactocin is associated with ethanol metabolism in Mycobacteria.  TABLE S1. Strains, plasmids, PCR primers used in the study | | |
| --- | --- | --- |
| Strain | **Description** | **Source** |
| *Escherichia coli* |  |  |
| DH5α | F- *φ80lacZΔM15* Δ(*lacZYA*-*argF*) U169 *recA1* *endA1* *hsdR17*(rk^-^, mk^+^) *phoAsupE44* *thi*-1 *gyrA96* *relA1* λ- | Invitrogen |
|  |  |  |
| *M. smegmatis* |  |  |
| mc^2^155 | High-frequency transformation mutant of *M. smegmatis* mc^2^6; ATCC^®^ 700084^™^ | (1) |
| ∆*mftA* | in-frame deletion mutant lacking 153 bp region of *mftA* (*MSMEG_1421*) | This study |
| ∆*mftB* | in-frame deletion mutant lacking 300 bp region of *mftB* (*MSMEG_1422*) | This study |
| ∆*mftC* | in-frame deletion mutant lacking 1169 bp region of *mftC* (*MSMEG_1423*) | This study |
| ∆*mftD* | in-frame deletion mutant lacking 1196 bp region of *mftD* (*MSMEG_1424*) | This study |
| ∆*mftE* | in-frame deletion mutant lacking 649 bp region of *mftE* (*MSMEG_1425*) | This study |
| ∆*mftF* | in-frame deletion mutant lacking 1403 bp region of *mftF* (*MSMEG_1426*) | This study |
| ∆*MSMEG_1410* | in-frame deletion mutant lacking 846 bp region of *MSMEG_1410* | This study |
| ∆*MSMEG_5866* | in-frame deletion mutant lacking 1122 bp region of *MSMEG_5866* | This study |
| ∆*MSMEG_6242* | in-frame deletion mutant lacking 1262 bp region of *MSMEG_6242* | This study |
| ∆*mftC*-Comp | Complemented *mftC* mutant carrying pMCpAINT::*mftC* at the *attB* site; Kan^R^ | This study |
|  |  |  |
| *M. marinum* |  |  |
| *M. marinum* | Laboratory stock of *M. marinum*  M strain |  |
| ∆*mftD_Mm_* | in-frame deletion mutant lacking 1167 bp region of *mftD* (*MMAR_1022*) | This study |
|  |  |  |
| *M. tuberculosis* |  |  |
| H37Rv | Laboratory stock of *M. tuberculosis* strain ATCC^®^ 27294^™^ |  |
| ∆*mftC_Mtb_* | *mftC* in-frame deletion mutant lacking 1168 bp region of *mftC* | This study |
| ∆*mftC_Mtb_*-Comp | Complemented *mftC* mutant carrying 2288 bp region of the *M. tuberculosis H37Rv* chromosome containing the *mftC* gene flanked by799 bp upstream and 307 bp downstream in the integrative vector pMCpAINT; Km^R^ | This study |
|  |  |  |
| Plasmids | **Description** | **Source** |
| p2NIL | *E. coli* cloning vector; (Kanamycin resistant - Km^R^) | (2) |
| pGOAL19 | Plasmid carrying *hyg, lacZ* and *sacB* genes as a *Pac*I cassette; (Hygromycin resistant - Hyg^R^; Ampicillin resistant - Ap^R^) | (3) |
| pMCpAINT | *E. coli*–Mycobacterium integrating shuttle vector; Km^R^ | (4) |
| p19∆*mftA* | *mftA* knockout vector. p2NIL sub clone carrying ∆*MSMEG_1421* gene – fusion of PCR products upstream (1398 bp) and downstream (1495 bp) of *MSMEG_1421*and *hyg-lacZ-sacB* cassette from pGOAL19; Km^R^ Hyg^R^ | This study |
| p19∆*mftB* | *mftB* knockout vector. p2NIL sub clone carrying ∆*MSMEG_1422* gene – fusion of PCR products upstream (1555 bp) and downstream (1503 bp) of *MSMEG_1422* and *hyg-lacZ-sacB* cassette from pGOAL19; Km^R^ Hyg^R^ | This study |
| p19∆*mftC* | *mftC* knockout vector. p2NIL sub clone carrying ∆*MSMEG_1423* gene – fusion of PCR products upstream (1506 bp) and downstream (1511 bp) of *MSMEG_1423* and *hyg-lacZ-sacB* cassette from pGOAL19; Km^R^ Hyg^R^ | This study |
| p19∆*mftD* | *mftD* knockout vector. p2NIL sub clone carrying ∆*MSMEG_1424* gene – fusion of PCR products upstream (1486 bp) and downstream (1296 bp) of *MSMEG_1424* and *hyg-lacZ-sacB* cassette from pGOAL19; Km^R^ Hyg^R^ | This study |
| p19∆*mftE* | *mftE* knockout vector. p2NIL sub clone carrying ∆*MSMEG_1425* gene – fusion of PCR products upstream (1494 bp) and downstream (1543 bp) of *MSMEG_1425* and *hyg-lacZ-sacB* cassette from pGOAL19; Km^R^ Hyg^R^ | This study |
| p19∆*mftF* | *mftF* knockout vector. p2NIL sub clone carrying ∆*MSMEG_1426* gene – fusion of PCR products upstream (1500 bp) and downstream (1429 bp) of *MSMEG_1426* and *hyg-lacZ-sacB* cassette from pGOAL19; Km^R^ Hyg^R^ | This study |
| p19∆*MSMEG_1410* | *MSMEG_1410* knockout vector. p2NIL sub clone carrying ∆*MSMEG_1410* gene – fusion of PCR products upstream (1423 bp) and downstream (1415 bp) of *MSMEG_1410* and *hyg-lacZ-sacB* cassette from pGOAL19; Km^R^ Hyg^R^ | This study |
| p19∆*MSMEG_5866* | *MSMEG_5866* knockout vector. p2NIL sub clone carrying ∆*MSMEG_5866* gene – fusion of PCR products upstream (1443 bp) and downstream (1495 bp) of *MSMEG_5866* and *hyg-lacZ-sacB* cassette from pGOAL19; Km^R^ Hyg^R^ | This study |
| p19∆*MSMEG_2687* | *MSMEG_2687* knockout vector. p2NIL sub clone carrying ∆*MSMEG_2687*gene – fusion of PCR products upstream (1443 bp) and downstream (1465 bp) of *MSMEG_2687* and *hyg-lacZ-sacB* cassette from pGOAL19; Km^R^ Hyg^R^ | This study |
| p19∆*MSMEG_6242* | *MSMEG_6242* knockout vector. p2NIL sub clone carrying ∆*MSMEG_6242* gene – fusion of PCR products upstream (1503 bp) and downstream (1445 bp) of *MSMEG_6242* and *hyg-lacZ-sacB* cassette from pGOAL19; Km^R^ Hyg^R^ | This study |
| pMCpAINT::*mftC* | *mftC* complementation vector. pMCpAINT harbouring PCR product of 2255 bp region of the *M. smegmatis* mc^2^155 chromosome containing the *mftC* gene flanked by771 bp upstream and 305 bp downstream sequences. | This study |
| p19∆*mftD_Mm_* | *mftD_Mm_* knockout vector. p2NIL sub clone carrying ∆*MMAR_1022* gene – fusion of PCR products upstream (1044 bp) and downstream (1497 bp) of *MMAR_1022* and *hyg-lacZ-sacB* cassette from pGOAL19; Km^R^ Hyg^R^ |  |
| p19∆*mftC_Mtb_* | *mftC_Mtb_* knockout vector. p2NIL sub clone carrying ∆*Rv0693* gene – fusion of PCR products upstream (1505 bp) and downstream (1503 bp) of *Rv0693* and *hyg-lacZ-sacB* cassette from pGOAL19; Km^R^ Hyg^R^ | This study |
| pMCpAINT::*mftC_Mtb_* | *mftC_Mtb_* complementation vector. pMCpAINT harbouring PCR product of 2288 bp region of the *M. tuberculosis* H37Rv chromosome containing the *mftC* gene flanked by 799 bp upstream and 307 bp downstream sequences. | This study |
|  |  |  |

1. Snapper, S. B., R. E. Melton, S. Mustafa, T. Kieser, and W. R. Jacobs, Jr**.** 1990. Isolation and characterization of efficient plasmid transformation mutants of *Mycobacterium smegmatis*. Mol Microbiol **4:**1911-9.
2. p2NIL was a gift from Tanya Parish Parish T, Stoker NG. 2000. Use of a flexible cassette method to generate a double unmarked *Mycobacterium tuberculosis tlyA* *plcABC* mutant by gene replacement. Microbiology **146**:1969-75.
3. Parish T, Stoker NG. 2000. Use of a flexible cassette method to generate a double unmarked *Mycobacterium tuberculosis tlyA* *plcABC* mutant by gene replacement. Microbiology **146**:1969-75.
4. Warner DF, Ndwandwe DE, Abrahams GL, Kana BD, Machowski EE, Venclovas Č, Mizrahi V. 2010. Essential roles for *imuA*′-and *imuB*-encoded accessory factors in DnaE2-dependent mutagenesis in *Mycobacterium tuberculosis*. Proceedings of the National Academy of Sciences **107**:13093-13098.

p2NIL (Addgene plasmid # 20188) and pGOAL19 (Addgene plasmid # 20190) was a gift from Tanya Parish. pMCpAINT was a gift from Digby Warner.

| **Oligonucleotides** | **Sequence (5´-3´)** | **Amplicon properties/ region targeted** |
| --- | --- | --- |
| **Oligonucleotides used for knockout vector construction^a^** | | |
| MSMEG_1421 UP F | *ggcgg***aagctt**aaatggctcgcgac | Forward and reverse primers used to PCR amplify 1401 bp+3 bp from 5′ end of *mftA* (*MSMEG_1421*) |
| MSMEG_1421 UP R | *ggcgg***ctcgag**catcgagtgccagaatgg |  |
| MSMEG_1421 DOWN F | *ggcgg***ctcgag**tgaccgtgtccactca | Forward and reverse primers used to PCR amplify 1498 bp +3 bp from 3′ end of *mftA* (*MSMEG_1421*) |
| MSMEG_1421 DOWN R | *ggcgg***ggatcc**tcacaccggactttcgttgc |  |
| MSMEG_1422 UP F | *ggcgg***aagctt**aaatggctcgcgac | Forward and reverse primers used to PCR amplify 1562 bp + 5 bp from 5′ end of *mftB* (*MSMEG_1422*) |
| MSMEG_1422 UP R | *ggcgg***ctcgag**gacacggtcagtagaccccgcac |  |
| MSMEG_1422 DOWN F | *ggcgg***ctcgag**atgacttcagttcagcccgtgcc | Forward and reverse primers used to PCR amplify 1512 bp + 3 bp from 3′ end of *mftB* (*MSMEG_1422*) |
| MSMEG_1422 DOWN R | *gacag***ggatcc**gacccatagcggtaccgcgc |  |
| MSMEG_1423 UP F | *ggcgg***aagctt**atcgacgcgaacacccccgtggac | Forward and reverse primers used to PCR amplify 1500 bp + 6 bp from 5′ end of *mftC* (*MSMEG_1423*) |
| MSMEG_1423 UP R | *ggcgg***ggatcc**agtcattgcgatccttcgggat |  |
| MSMEG_1423 DOWN F | *ggcgg***ggatcc**tgtgaacatggcacgagacatctgg | Forward and reverse primers used to PCR amplify 1500 bp + 5 bp from 3′ end of *mftC* (*MSMEG_1423*) |
| MSMEG_1423 DOWN R | *ggcgg***aagctt**gaccacggtgccggccacagctg |  |
| MSMEG_1424 UP F | *ggcgg***aagctt**gtgtccactcaggt | Forward and reverse primers used to PCR amplify 1495 bp + 5 bp from 5′ end of *mftD* (*MSMEG_1424*) |
| MSMEG_1424 UP R | *gacgc***gcggccgc**gttcacaccggactttc |  |
| MSMEG_1424 DOWN_F | *ggcgg***gcggccgc**gtgaggtcctcccgcggggc | Forward and reverse primers used to PCR amplify 1301 bp + 3 bp from 3′ end of *mftD* (*MSMEG_1424*) |
| MSMEG_1424 DOWN R | *gagacg***ggatcc**ccgttgctgcgggtgtgccg |  |
| MSMEG_1425 UP F | *ggcgg***aagctt**ggtgaccactcgcggggcac | Forward and reverse primers used to PCR amplify 1503 bp + 3 bp from 5′ end of *mftE* (*MSMEG_1425*) |
| MSMEG_1425 UP R | *ggcgg***tttaaa**catgctgtgtagctgcctcgac |  |
| MSMEG_1425 DOWN F | *ggcgg***tttaaa**cggaacggattgctgacatgacc | Forward and reverse primers used to PCR amplify 1534bp + 21bp from 3′ end of *mftE* (*MSMEG_1425*) |
| MSMEG_1425 DOWN R | *gagcaat***ggatcc**gacggggccgggccggcctc |  |
| MSMEG_1426 UP F | *gatgccc***aagctt**gaggcgctgaccaag | Forward and reverse primers used to PCR amplify 1512 bp + 6 bp from 5′ end of *mftF* (*MSMEG_1426*) |
| MSMEG_1426 UP R | *ggcgg***ctcgag**ggtcatgtcagcaatccgttc |  |
| MSMEG_1426 DOWN F | *ggcgg***ctcgag**ttagccgccgactttgag | Forward and reverse primers used to PCR amplify 1437 bp + 4 bp from 3′ end of *mftF* (*MSMEG_1426*) |
| MSMEG_1426 DOWN R | *ggcgg***ggtacc**tcaggtcgcgatgaactcg |  |
| MSMEG_6242 UP F | *ggcgg***aagctt**atgcgacggcggcgttgaacgtcggcgc | Forward and reverse primers used to PCR amplify 1500 bp + 3 bp from 5′ end of *MSMEG_6242* |
| MSMEG_6242 UP R | *gcggc***agatct**cattggttcactcctcgctgtg |  |
| MSMEG_6242 DOWN F | *ggcgg***agatct**tgattcacgtctgattcgcaaccg | Forward and reverse primers used to PCR amplify 1452 bp + 3 bp from 3′ end of *MSMEG_6242* |
| MSMEG_6242 DOWN R | *ggcgg***aagctt**cgacctcggccgcgcgttcgttgtac |  |
| MSMEG_2687 UP F | *ggcgg***aagctt**catcgggcccgagcccggccacgt | Forward and reverse primers used to PCR amplify 1440 bp + 3 bp from 5′ end of *MSMEG_2687* |
| MSMEG_2687 UP R | *ggcgg***ggtacc**catatgtgggtcctgacggaagtggtc |  |
| MSMEG_2687 DOWN F | *ggcgg***ggtacc**tgaaccaggaggccgcatgagcatc | Forward and reverse primers used to PCR amplify 1462bp + 3 bp from 3′ end of *MSMEG_2687* |
| MSMEG_2687 DOWN R | *ggcgg***aagctt**ccgcggtgccgagggccgcgtgatcg |  |
| MSMEG_1410 UP F | *ggcgg***aagctt**cgggcagaggccgtgctgttcggc | Forward and reverse primers used to PCR amplify 1420 bp + 3 bp from 5′ end of *MSMEG_1410* |
| MSMEG_1410 UP R | *ggcgg***ggtacc**catccccggtcctttctcgatg |  |
| MSMEG_1410 DOWN F | *ggcgg***ggtacc**tgagttcccgccagaaatctctcgc | Forward and reverse primers used to PCR amplify 1412bp + 3 bp from 3′ end of *MSMEG_1410* |
| MSMEG_1410 DOWN R | *ggcgg***aagctt**gtgaactcgcacaccatgacccc |  |
| MSMEG_5866 UP F | *ggcgg***aagctt**caccacaccagttcgggcaccgcg | Forward and reverse primers used to PCR amplify 1440 bp + 3 bp from 5′ end of *MSMEG_5866* |
| MSMEG_5866 UP R | *ggcgg***ggtacc**catcaaagctcctgctttccg |  |
| MSMEG_5866 DOWN F | *ggcgg***ggtacc**tgacaggggatccatgaccg | Forward and reverse primers used to PCR amplify 1492bp + 3 bp from 3′ end of *MSMEG_5866* |
| MSMEG_5866 DOWN R | *ggcgg***aagctt**ttggtgggaggtggccgacc |  |
| MMAR_1022 UP F | *ggcgg***ggatcc**gggtgagttgtccacccggcaatgcc | Forward and reverse primers used to PCR amplify 1041 bp + 3 bp from 5′ end of *mftD_Mm_* (*MMAR_1022)* |
| MMAR_1022 UP R | *ggcgg***aagctt**catgactacacgggactttcgttgcac |  |
| MMAR_1022 DOWN F | *ggcgg***aagctt**taggaatccgggcctgccttacc | Forward and reverse primers used to PCR amplify 1492bp + 3 bp from 3′ end of *MSMEG_5866* |
| MMAR_1022 DOWN R | *ggcgg***ggatcc**cggcgccaggcccacaatgcgaggcgcg |  |
| Rv0693 UP F | *ggcgg***aagctt**ttggaggcaggcgatgagaagtcc | Forward and reverse primers used to PCR amplify 1500 bp + 5 bp from 5′ end of *mftC* (*Rv0693*) |
| Rv0693 UP R | *ggcgg***agatct**gtcatgttgtctgccgaggaaccagc |  |
| Rv0693 DOWN F | *ggcgg***agatct**tagccgtggccgaagcgtggtttg | Forward and reverse primers used to PCR amplify 1500 bp + 3 bp from 5′ end of *mftC* (*Rv0693*) |
| Rv0693 DOWN R | *ggcgg***aagctt**gctgctcggtggaccccagcggg |  |
| **Oligonucleotides used for genetic complementation vector construction** | | |
| Rv0693_Compl. New F | *ggcgg***aagctt**gcggccgcggcccgcgcgac | Forward and reverse primers used to PCR amplify 2288 bp region of the  *M. tuberculosis* H37Rv chromosome containing the *mftC* gene flanked by 799 bp upstream and 307 bp downstream sequences. |
| Rv0693_Compl. New R | *ggcgg***aagctt**ttcggattcgtcaaaggtgttg |  |
| MSMEG_1423 Compl. New F | *ggcgg***aagctt**cagggcgtcgccgagtgacacctc | Forward and reverse primers used to PCR amplify 2255 bp region of the *M. smegmatis* mc2155 chromosome containing the *mftC* gene flanked by771 bp upstream and 305 bp downstream sequences. |
| MSMEG_1423 Compl. New R | *ggcgg***aagctt**ggcggcccgagcaaccgcgacctcgccg |  |
| **Oligonucleotides used for PCR-based genotyping** | | |
| MSMEG_1421-22-23 Screen F | cagggcgtcgccgagtgacacctc | Forward and reverse primers used for PCR-based  genotyping of the *mftA,B*,*C* allele. ∆*mftA*: 2102 bp; ∆*mftB*: 1949 bp; ∆*mftC*: 1081 bp; WT: 2255 bp. |
| MSMEG_1421-22-23 Screen R | ggcggcccgagcaaccgcgacctcgccgt |  |
| MSMEG_1424 Screen F | acttcgacgcctgccgcggtggctgcatgg | Forward and reverse primers used for PCR-based  genotyping of the *mftD*. ∆*mftD*: 500 bp; WT: 1787 bp. |
| MSMEG_1424 Screen R | ccaccggaggggccaccaccgcgtcgcggtc |  |
| MSMEG_1425 Screen F | gatcggacgcgcctacctgtggggtctggc | Forward and reverse primers used for PCR-based  genotyping of the *mftE*. ∆*mftE*: 679 bp; WT: 1318 bp. |
| MSMEG_1425 Screen R | ctgtgacgtccagatgggacggcccgctgg |  |
| MSMEG_1426 Screen F | ggtgacccgacgaccgcgacagccgccgaagg | Forward and reverse primers used for PCR-based  genotyping of the *mftF*. ∆*mftF*: 679 bp; WT: 1986 bp. |
| MSMEG_1426 Screen R | cagccgtcgaattcgtggacccggcgcacc |  |
| MSMEG_5866 Screen F | ggcagcgcgacttcttcgacttcggtcacg | Forward and reverse primers used for PCR-based  genotyping of the *MSMEG_5866*. ∆*MSMEG_5866*: 399 bp; WT: 1515 bp. |
| MSMEG_5866 Screen R | cggccatcagggccagccagccctcccgg |  |
| MSMEG_1410 Screen F | gcatccgtgcgaccgggatcccggcg | Forward and reverse primers used for PCR-based  genotyping of the *MSMEG_1410*. ∆*MSMEG_1410*: 750 bp; WT: 1590 bp. |
| MSMEG_1410 Screen R | gcatgtatccaccgctggtcgggttgagca |  |
| MSMEG_6242 Screen F: | ggcaaccggatcgccgtgcgcttcgcctac | Forward and reverse primers used for PCR-based  genotyping of the *MSMEG_6242*. ∆*MSMEG_6242*: 700 bp; WT: 1964 bp. |
| MSMEG_6242 Screen R | ggttgtcgtcgagaccctcgtagcactgc |  |
| MSMEG_2687 Screen F | gcacgagcacgacggcacgttcgtcttcg | Forward and reverse primers used for PCR-based  genotyping of the *MSMEG_2687*. ∆*MSMEG_2687*: 809 bp; WT: 1625 bp. |
| MSMEG_2687 Screen R | gccgacaacgtgaccgatccgacgcgcatc |  |
| MMAR_1022 Screen F | gggtgagttgtccacccggcaatgcc | Forward and reverse primers used for PCR-based  genotyping of the *mftD_Mm_* (*MMAR_1022)*. ∆ *mftD_Mm_*: 1367 bp; WT: 2568bp. |
| MMAR_1022 Screen R | tcgcgatccgggtgtcggtatccaacggc |  |
| **Oligonucleotides used for probe generation for southern blot analysis** | | |
| MSMEG_1421_22 Southern F | gtggcacgcggccacgtttcatggcgcagacgc | 690 bp amplicon spanning from the 3’- end of *MSMEG_1419* to within *MSMEG_1420*; used as a probe to genotype ∆*mftA* and ∆*mftB* by southern blot (see FIG S1). |
| MSMEG_1421_22 Southern R | cgcggggcgtcgccgctccaccacgcaggacc |  |
| MSMEG_1423 Southern F | gatcggacgcgcctacctgtggggtctggc | 1980 bp amplicon from the 3’- end of *MSMEG_1425* to within *MSMEG_1428;* used as a probe to genotype ∆*mftC*, ∆*mftD*, and ∆*mftE* by southern blot (see FIG S1) |
| MSMEG_1423 Southern R | ctgtgacgtccagatgggacggcccgctgg |  |
| MSMEG_1426 Southern F | gccgctgctcggcctgcgcctcacggcattcc | 519 bp amplicon within *MSMEG_1427;* used as a probe to genotype ∆*mftF* by southern blot (see FIG S1). |
| MSMEG_1426 Southern R | ccggcggatcacccgcagctgttgcccgtcgcg |  |
| MSMEG_1410 Southern F: | ggactggctgctggaccgcaagatcacgcc | 880 bp amplicon within *MSMEG_1409*; used as a probe to genotype ∆*MSMEG_1410* by southern blot (see FIG S1). |
| MSMEG_1410 Southern R | cagcagcgaattggtgtgcgtgttgcggtcgtacg |  |
| MSMEG_5866 Southern F | gaccgaagcgaccgagacttccaccgagac | 1090 bp amplicon spanning from the 5’- end of *MSMEG_5867* to within *MSMEG_5869;* used as a probe to genotype ∆*MSMEG_5866* by southern blot (see FIG S1). |
| MSMEG_5866 Southern R | gccgcggcgtcgagcgcgccctgggcgctc |  |
| MSMEG_6242 Southern F | ggcgtcggcaagaccgaactggccaaggcg | 871 bp amplicon within *MSMEG_6241*; used as a probe to genotype ∆*MSMEG_6242* by southern blot (see FIG S1). |
| MSMEG_6242 Southern R | gcccgcggaacaccgcggtggtcgtgttgg |  |
| Rv0693_Southern F | ccggtgcaatccgccgagcagccgaagcggcacc | 770 bp amplicon within *MSMEG_6241* (see FIG S1). |
| Rv0693_Southern R | gggccgacggcgctcaaccacaccgcaccac |  |
| **Oligonucleotides used for qRT-PCR** | | |
| MSMEG_6242_f | cgcaacatcaacgagttc |  |
| MSMEG_6242_r | gtgtcggtgatgacgtag |  |
| MSMEG_1423_f | cagtgcaaggacatcatc |  |
| MSMEG_1423_r | gttggtggagaacttgac |  |
| SigA_F | tgccgatctgcttgaggtagg |  |
| SigA_R | ttcgtgtgggacgaggaagag |  |
| a. GC-clamp sequences (non-H37Rv) are italicized; Restriction sites are shown in bold | | |
