# Supplementary material for: Mycofactocin Is Associated with Ethanol Metabolism in Mycobacteria
Source: mBio. 2019 May 21;10(3):e00190-19. doi: 10.1128/mBio.00190-19 (PMC6529628; doi:10.1128/mBio.00190-19)
Supplement: TABLE S3 [file mBio.00190-19-st003.doc]

**Gopinath Krishnamoorthy, Peggy Kaiser, Laura Lozza, Karin Hahnke, Hans-Joachim Mollenkopf, Stefan H. E. Kaufmann. Mycofactocin is associated with ethanol metabolism in Mycobacteria.**

**Table S3: Gene expression values from Cholesterol:EtOH treated-∆*mftC_Mtb_* relative to untreated cells.**

| **Gene name/ locus name** | **Gene annotation** | **Fold changes** |
| --- | --- | --- |
| **Central carbon metabolism** | | |
| *pdhA* | pyruvate dehydrogenase A | -1.99 |
| *pdhB* | pyruvate dehydrogenase B | -2.329 |
| *pdhC* | pyruvate dehydrogenase C | -2.102 |
| **Redox** | | |
| *qcrA* | putative Rieske iron-sulfur protein QcrA | -2.169 |
| *rubA* | probable rubredoxin rubA | 3.1876 |
| *rubB* | probable rubredoxin rubB | 3.1885 |
| *whiB2* | transcriptional regulatory protein whiB2 | -4.292 |
| *whiB3* | transcriptional regulatory protein whiB3 | 2.4392 |
| **Bioenergetics** | | |
| *atpE* | ATP synthase C chain | -1.5 |
| *atpF* | ATP synthase B chain | -1.527 |
| *atpH* | ATP synthase delta chain | -1.683 |
| *cydA* | cytochrome D ubiquinol oxidase (subunit I) | -1.641 |
| *nadA* | probable quinolinate synthetase | -1.833 |
| *nadB* | probable L-aspartate oxidase | -2.065 |
| *nadC* | probable nicotinate-nucleotide pyrophosphatase | -2.494 |
| **DosR regulon** | | |
| *Rv0079* | hypothetical protein | 2.1118 |
| *Rv0569* | hypothetical protein | 1.9633 |
| *Rv1733c* | putative transmembrane protein | 3.6671 |
| *Rv1737c* | possible nitrate/nitrite transporter *nark2* | 2.3104 |
| *Rv1738* | hypothetical protein | 4.3644 |
| *Rv2031c* | heat shock protein *hspx* | 2.9799 |
| *Rv2032* | hypothetical protein *acg* | 1.9035 |
| *Rv2627c* | hypothetical protein | 2.8596 |
| *Rv2628* | hypothetical protein | 2.5305 |
| *Rv3127* | hypothetical protein | 2.8852 |
| *Rv3130c* | hypothetical protein | 4.5647 |
| *Rv3131* | hypothetical protein | 6.4005 |
| *Rv3134c* | hypothetical protein | 2.9486 |
| *tgs1* | Triacylglycerol synthase | 4.3529 |
| **Dehydrogenase** | | |
| *Rv0223c* | putative aldehyde dehydrogenase | 3.1936 |
| *Rv1895* | putative dehydrogenase | 2.3551 |
| *Rv1896* | putative dehydrogenase | 2.2778 |
| **Cholesterol metabolism** | | |
| *cyp125* | Probable cytochrome p450 | 3.7071 |
| *cyp132* | Putative cytochrome P450 | 3.9503 |
| *cyp142* | Probable cytochrome p450 monooxygenase | 2.5466 |
| *fadA5* | Acetyl CoA acetyltransferase | 6.4253 |
| *fadA6* |  | 10.37 |
| *fadD18* | Fatty acid CoA ligases | 1.8205 |
| *fadD19* |  | 2.0704 |
| *fadD3* | putative fatty-acid-CoA ligase fadD3 | 3.1978 |
| *fadE26* | Acyl CoA dehydrogenases | 1.6851 |
| *fadE27* |  | 1.8667 |
| *fadE28* |  | 3.3008 |
| *fadE29* |  | 2.714 |
| *fadE30* |  | 4.4203 |
| *fadE31* |  | 3.0072 |
| *fadE32* |  | 2.0526 |
| *fadE33* |  | 3.6373 |
| *hsaA* | Possible oxidoreductases involved in cholesterol catabolism | 3.8874 |
| *hsaB* |  | 1.7873 |
| *hsaC* |  | 3.7952 |
| *hsaD* |  | 3.297 |
| *hsaF* |  | 1.7725 |
| *hsaG* |  | 1.5441 |
| *icl* | Isocitrate lyase | 9.256 |
| *kshA* | ketosteroid-9-alpha-hydroxylase. | 3.5471 |
| *kshB* | ketosteroid-9-alpha-hydroxylase. | 2.0922 |
| *kstD* | Probable dehydrogenase | 2.1175 |
| *kstR* | Transcriptional regulatory protein | 1.5441 |
| *ltp2* | Lipid transfer proteins | 2.8519 |
| *ltp3* |  | 1.6816 |
| *ltp4* |  | 2.6624 |
| *Rv1129c* | Transcriptional regulator | 2.3656 |
| *Rv1130* | *prpD* | 13.223 |
| *Rv1131* | *prpC* | 14.27 |
| *Rv1132* | hypothetical protein | 2.0884 |
| *Rv3502c* | putative short-chain type dehydrogenase/reductase | 1.8711 |
| *Rv3531c* | hypothetical protein | 2.3988 |
| *Rv3538* | Possible 2-enoyl acyl-CoA hydratase. | 2.6364 |
| *Rv3541c* | Conserved hypothetical protein | 3.0134 |
| *Rv3542c* | Conserved hypothetical protein | 3.9379 |
| *Rv3547* | Conserved hypothetical protein | 1.9355 |
| *Rv3548c* | putative short-chain type dehydrogenase/reductase | 4.8368 |
| *Rv3549c* | putative short-chain type dehydrogenase/reductase | 4.8797 |
| *Rv3551* | putative CoA-transferase subunit alpha | 4.6609 |
| *Rv3552* | putative CoA-transferase subunit beta | 10.21 |
| *Rv3553* | Oxidoreductase | 6.0062 |
| *Rv3559c* | Oxidoreductase | 6.3056 |
| **Growth and cell division** | | |
| *fas* | Fattyacid synthase | -2.318 |
| *ftsK* | Cell division protein | -2.497 |
| *ftsZ* | Cell division protein | -2.778 |
| *kasA* | Beta-ketoacyl-ACP synthase | -3.01 |
| *kasB* | Beta-ketoacyl-ACP synthase | -2.935 |
| *ripA* | Peptidoglycan hydrolase | -2.217 |
| *rpfC* | Resuscitation-promoting factor *rpfC* | -1.793 |
| *Rv2163c* | penicillin-binding membrane protein pbpB | -2.262 |
| *Rv2164c* | Conserved proline rich membrane protein | -2.422 |
| *Rv2165c* | hypothetical protein | -1.713 |
| *Rv2166c* | hypothetical protein | -8.063 |
| *wag31* | Wag31 | -1.644 |
